# Plasma Pretreatment of Pt Single-Atom Precursors Supported on Mechanically Activated Al_2_O_3_: Enhanced Performance in Propane Dehydrogenation

**DOI:** 10.1021/jacs.5c12230

**Published:** 2025-10-27

**Authors:** Jingyi Yang, Eduardo Ortega, Joonbaek Jang, Andrea Martini, Jie Zhu, Janis Timoshenko, Jacopo De Bellis, Ferdi Schüth, Shamil Shaikhutdinov, Beatriz Roldan Cuenya

**Affiliations:** † Department of Interface Science, 28259 Fritz-Haber-Institut der Max-Planck-Gesellschaft, Faradayweg 4-6, Berlin 14195, Germany; ‡ Department of Heterogeneous Catalysis, 28314 Max-Planck-Institut für Kohlenforschung, Kaiser-Wilhelm-Platz 1, Mülheim an der Ruhr 45470, Germany

## Abstract

We investigated the effect of catalyst pretreatment with “cold” plasmas on the catalytic performance of Pt single-atom catalyst (SAC) precursors supported on Al_2_O_3_ for the propane dehydrogenation reaction. We found that the catalysts treated with a hydrogen plasma before the conventional calcination/reduction steps showed considerably increased propane conversion without loss of selectivity, whereas exposure to an argon plasma did not result in such an effect. Moreover, the H_2_ plasma-treated catalyst showed a lower deactivation rate, suggesting better long-term stability. The structural and chemical evolution of the catalysts studied by TEM, XAS, XPS, and DRIFTS showed that the H_2_ plasma: (i) partially reduces singly dispersed Pt^2+^ species and promotes Pt clustering; (ii) decreases the amount of Cl remaining after the use of the hexachloroplatinic acid precursor; and (iii) enhances surface defects in the alumina support. We propose that the promotional effect of the H_2_ plasma lies in the specific modification of the alumina surface, which in turn alters the metal-support interaction and leads to the formation of a more active Pt/Al_2_O_3_ interface during the subsequent oxidation and reduction steps. The results show that nonthermal plasma treatments of single-atom precursors can become a tool for tuning the catalytic performance of highly dispersed metal catalysts.

## Graphical abstract

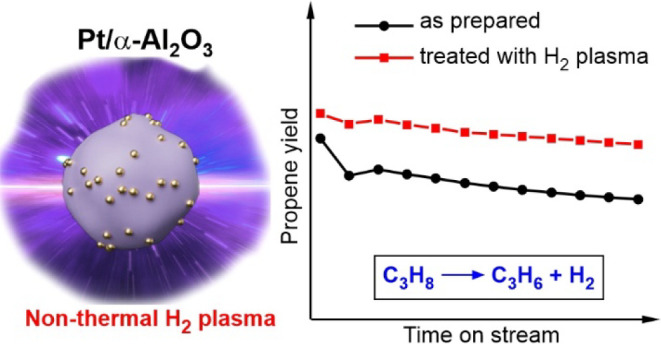

## 1 Introduction

Catalysis by single atoms is a rapidly growing field in heterogeneous catalysis. Initially driven by attempts to maximize noble metal usage efficiency, several studies have shown that single atoms may exhibit intrinsic activity higher than “conventional” metal nanoparticles (NPs), especially in oxidation reactions.^1–5^ However, the implementation of single-atom catalysts (SACs) critically depends on the stability of the single atoms (SAs) toward sintering. Since the latter involves metal atom diffusion across the support surface, any chemical or physical modification of the support should alter the metal sintering process and, hence, the stability of the SACs. To verify this approach, we have recently employed a low-temperature (“cold”) plasma for the SAC pretreatment.^6^ Certainly, the interaction of the plasma with the catalyst surface is a very complex process, since the plasma contains a variety of highly reactive species, which may cause the formation of various functional groups as well as structural defects, all at near room temperature.^7–13^ Nonetheless, we found that catalyst pretreatment with an oxygen plasma improved the stability and reactivity of Pt/CeO_2_ SACs in the CO oxidation reaction.^6^ On the other hand, SAs are ideal precursors for generating uniform metal nanoparticles with a narrow size distribution, as they provide a homogeneous molecular structure and might allow one to retain a strong functional interface with the support upon suitable activation. Slow aggregation and a high abundance of nucleation sites created by plasma could favor the formation of NPs with a raft-like morphology.

Inspired by these ideas, here we focused on the implementation of cold plasmas to SACs in reactions taking place in reducing (H_2_ containing) atmospheres. More specifically, we addressed the reaction of propane dehydrogenation to propene (PDH), which is a highly demanding process in industry and currently employs Pt-based catalysts at temperatures of 500–600 °C.^14–17^ Previous studies showed that the PDH reaction over Pt catalysts is structure sensitive^18^ and often exhibits particle size effects, such that the turnover frequency is higher for the smaller particles.^19,20^ Nonetheless, despite being considered initially active, the Pt SACs were found to be unstable under reaction conditions, probably because of the sintering of the SAs into clusters and NPs in the reducing environment.^21^ In this respect, so-called “fully exposed cluster catalysts”, as a bridge linking SAs and NPs, have been invoked as effective catalysts for alkane dehydrogenation reactions.^22^ However, there is still no full understanding of the transformations that the SACs undergo during the reaction and their correlation with the catalytic performance.

Here, we propose to make use of the controlled aggregation of the SACs and consider them as precursors for generating stable and small metal aggregates with controllable morphological and, hence, electronic properties. Retaining a large functional interface between the support and the active metal provides a means for better stability of active nanostructures, even under harsh conditions of operation and regeneration.

The catalytic performance of Pt-based catalysts is affected by the chemical nature of the functional interface, as well the choice and defect structure of the oxide support. Alumina (Al_2_O_3_) is the most studied oxide support due to its potentially high surface area and thermal stability.^15,23–25^ In order to improve the interaction with Pt and ensure its high dispersion, modified alumina supports have been proposed for the preparation of highly active Pt-based catalysts. In particular, it was emphasized the important role of penta-coordinated Al^3+^ species, which stabilized raft-like Pt particles.^23^ Here, we introduce mechanically activated (ball-milled) α-Al_2_O_3_ as a support.^26^ Alumina prepared by the ball milling method exhibits a high surface area, and the mechanochemical synthetic approach also induces defects that might play a role in the reactivity and stability of the Pt/Al_2_O_3_ SACs. Since the α-phase is thermodynamically the most stable one, it is not expected that substantial changes to the support’s surface and bulk structure would occur during the harsh operating conditions of PDH, this being a prerequisite for the stability of the functional interface and, hence, for the enhanced long-term stability of the noble metal catalyst.

In this study, we addressed the question of whether a plasma pretreatment can improve the catalytic performance of the Pt/Al_2_O_3_ SACs in the PDH reaction, bearing in mind the rather weak interaction of Pt with the α-Al_2_O_3_ surface.^27^ The idea is to use plasma as a low-temperature method to transform the Pt SAC precursors into Pt raft particles of small size (with a strong functional interface) by creating additional defect sites on the Al_2_O_3_ support for later occupation by Pt during conventional oxidation/reduction treatments. Moreover, we employed the α-Al_2_O_3_ support prepared by ball milling^28,29^ to benefit from the refractory nature of the stable α-phase of the bulk support and simultaneously bind the Pt precursor to defect sites that should be present after the mechanochemical synthesis. In this respect, we further followed a recent PDH study on Pt/Al_2_O_3_ catalysts prepared by ball milling^30^ and used them as a benchmark to elucidate the plasma effects on both the Pt species and the Al_2_O_3_ support. In this reference study, stable catalytic performance at a low temperature (500 °C) and a low deactivation rate were associated with small (2 nm) Pt particles formed upon ball-milling-assisted synthesis. The question we address here is whether a plasma treatment can further improve prior performance.

The PDH reaction is an endothermic process that operates at high temperatures. Under these conditions, a Pt catalyst capable of binding propane would also preferentially dehydrogenate the product (propene) and activate hydrogen. The latter may initiate hydrogenolysis and coke formation, which, in turn, deactivates the catalyst. The intrinsic activity of Pt is too high to achieve high selectivity,^31^ even when the reaction conditions are chosen to favor the rapid transport of the product. In addition, the PDH reaction on Pt is structure-sensitive: the Pt(100) surface is more active but less selective than Pt(111).^18^ Moreover, less coke is predicted to form on the (111) surface. Thus, a strongly binding functional interface should facilitate the formation of Pt particles that preferentially expose the (111) surface while keeping the particles small enough to prevent/minimize graphene formation on their surface.^32^

According to a prior study,^33^ atomic hydrogen activates the alumina surface by chemically removing oxygen ions, thereby creating coordinatively unsaturated Al ions. However, it remains to be shown if these species or hydroxyl species, resulting from the reaction of the initial unstable Al–H species with traces of oxygen in the environment, are the active sites for binding Pt. In any case, the Pt atoms resulting from the chemical reduction of the ionic SAs should be incorporated into the environment of activated alumina. Plasma treatment here serves as a means of restructuring the stable surface termination of α-Al_2_O_3_ by creating Al species in lower coordination environments than the octahedral ones existing in the ideal structure. In order to discriminate the pure restructuring effect of the plasma treatment from an additional chemical reduction effect induced by the H_2_ plasma, we compared conventionally prepared catalyst samples with samples pretreated with Ar or H_2_ plasmas. Here, we considered that the plasma treatment not only affects the formation of the Pt species, which constitute a tiny minority of the catalyst surface, but also modifies the bare support.

## 2 Materials and Methods

The Al_2_O_3_ support was prepared by a ball-milling technique, as described in detail elsewhere,^29^ using boehmite (γ-AlOOH, from Sasol) as the precursor. The milling equipment (Fritsch Planetary Micro Mill P7 Pulverisette) included custom-made corundum milling jars and grinding balls. For the synthesis, 1 g of γ-AlOOH was placed in a 45 mL corundum jar and milled at 700 rpm for 12 h using balls (10 mm in diameter) made of the same material. After 6 h of milling, the powder was mobilized by scratching it off from the inner jar walls and balls; milling was then reinitiated until the program was completed. For each repetition of the synthesis, new balls were used to minimize abrasion. Finally, the Al_2_O_3_ powder was calcined at 600 °C in air for 10 h.

For the preparation of the Pt/Al_2_O_3_ catalysts, 1 g of Al_2_O_3_ was dispersed in 40 mL of ethanol, and an aqueous solution containing 40 μmol/mL of H_2_PtCl_6_ (Sigma-Aldrich) was added dropwise. The obtained mixture was stirred at room temperature for 4 h, centrifuged, and thoroughly washed with ethanol. Subsequently, the sample was dried under vacuum at 60 °C overnight. The Pt loading, determined by inductively coupled plasma mass spectrometry, agreed with the nominal value of 0.2 wt %. The 0.2 wt % Pt/Al_2_O_3_ catalysts were only investigated in this study.

Catalytic tests were carried out in a fixed-bed reactor at atmospheric pressure. The fresh catalyst (100 mg, 100–200 μm mesh) was loaded into the quartz tube reactor (inner diameter, 4.5 mm). The sample was calcined in 20 vol % O_2_/Ar flow at 200 °C for 30 min (heating rate 3 °C/min) and reduced at 540 °C in 10 vol % H_2_/Ar flow for 10 min (heating rate 2 °C/min). For the PDH reaction, the mixture of 80% C_3_H_8_ + 10% H_2_ + 10% Ar was used at a flow rate of 6 mL/min, which corresponds to a propane-based weight hourly space velocity (WHSV) of 5.7 h^–1^.

The propane conversion (*X*_C3H8_) and selectivity to propene (*S*_C3H6_) were calculated based on the sum of all detected carbon-containing products:

$$X_{C3H8}=\frac{\overset{n}{\underset{i}{\sum }}N_{i}c_{i}}{3c_{C3H8,out}+\overset{n}{\underset{i}{\sum }}N_{i}c_{i}}\times 100$$

$$S_{C3H6}=\frac{3c_{C3H6}}{\overset{n}{\underset{i}{\sum }}N_{i}c_{i}}\times 100$$

Plasma treatments were performed in a homemade setup, schematically shown in Figure S1 in the Supporting Information. Plasma was generated by applying A.C. voltage (30 kV, 40 kHz) between a silica-coated W inner electrode and an outer Cu electrode, with the root-mean-square (RMS) power set to 200 W for all samples studied. The gas (Ar and H_2_) pressure was typically about 5 mbar.

Pt dispersion in the reduced catalysts was determined via pulsed CO uptake measurements using an Autosorb-1C chemisorption setup equipped with an IR detector. The samples were heated to 120 °C in He flow and then in 10 vol % H_2_/He at 400 °C for 2 h. The samples were cooled to room temperature in He and subsequently exposed to pulses of CO until saturation. The same procedure was applied to the benchmark Pt/Al_2_O_3_ sample (QC-Standard from 3P Instruments) with 100% dispersion.

Scanning Transmission Electron Microscopy (STEM) characterization was performed using a probe-corrected JEM-ARM 200F microscope (from JEOL) operated at 200 kV. The high-angle annular dark field (HAADF) and bright field (BF) images were collected at 50–180 and 30 mrad collection semiangles, respectively, with a nominal e-beam current of 10 pA.

X-ray absorption spectroscopy (XAS) spectra were collected at the BL22-CLAESS end-station at the ALBA synchrotron (Spain), which is equipped with a Si(311) double-crystal monochromator. Measurements at the Pt L_3_-edge were performed in fluorescence mode using an energy-selective six-channel SDD detector. Measurements of reference samples were performed in transmission mode by using ionization chambers as detectors. Ionization chambers were filled with N_2_:Kr mixtures at 96:4, 35:65, and 0:100 ratios for *I*_0_, *I*_1_, and *I*_2_ detectors, respectively. The X-ray energy was calibrated using Pt foil. *Operando* XAS measurements were performed using an in-house-built capillary reactor, featuring a quartz capillary with an outer diameter of 1 mm (inner diameter 0.9 mm) and an electrical heating element. The temperature was measured using a thin thermocouple inserted into the capillary. All measurements were performed at 1 bar of gas pressure. The X-ray absorption near-edge structure (XANES) and extended X-ray absorption fine structure (EXAFS) spectra were first collected at room temperature in He flow, followed by *in situ* measurements during the reduction in a 20 vol % H_2_/He mixture with a total flow rate of 20 mL/min, while the temperature was ramped to 540 °C at a rate of 2 °C/min. Afterward, the gas feed was switched to the reaction mixture (C_3_H_8_:H_2_:He, with a 20:1:4 molar ratio) at a total flow rate of 25 mL/min and kept under reaction conditions for at least 1 h. The sample was then cooled in He to room temperature to collect high-quality EXAFS spectra.

The ATHENA and ARTEMIS software packages were employed to process the XAS spectra.^34^ First-shell EXAFS fitting was performed in *R*-space in the range between 1.2 and 3.3 Å by using a *k*-weighting of 2. For the Fourier transform, a *k*-range of 3.0 to 9.0 Å^–1^ was used for the high-temperature data, while a longer *k*-range of 3.0 to 12.0 Å^– 1^ was applied to the room-temperature data with better signal quality. In the fitting, we considered contributions from the nearest-neighbor Pt–Pt, Pt–Cl, and Pt–O bonds. Amplitude reduction (*S*_0_^2^) factors obtained using Pt foil, H_2_PtCl_6_, and H_2_PtOH_6_ reference spectra for Pt–Pt, Pt–Cl, and Pt–O contributions were 0.79, 0.77, and 0.81, respectively. For each path, its coordination number, bond length, and disorder factor σ^2^ were treated as fitting variables. In addition, a correction to the photoelectron reference energy Δ*E*_0_, common for all scattering paths, was refined. Disorder factors (σ^2^ values) for the high temperature data were assumed to be the same for all catalysts at a given temperature. C_3_ cumulant values accounting for the skewness of the pair distribution function at high temperatures were fixed and taken from ref 35.

X-ray Photoelectron Spectroscopy (XPS) spectra were measured in an ultrahigh vacuum (UHV) chamber (from Specs) equipped with a Phoibos 150 analyzer and a monochromatic Al K_α_ X-ray source (*h*ν = 1486.6 eV). The powder catalyst samples were pressed into a stainless steel sample holder with a 0.5 mm recess of ∼6 mm in diameter and transferred into the UHV chamber via a load lock. To compensate for sample charging, we employed a flood gun FG-20 (Specs). A plasma treatment at UHV-compatible pressures was performed using a plasma source (OSPrey, Oxford Scientific) operated at 10^–4^ mbar of H_2_, an anode voltage of 750 V, and an emission current of 1.5 μA. Oxidation and reduction treatments at near-atmospheric pressures were performed in an HPC 20 high-pressure cell (Specs) connected to the main chamber via a gate valve. The sample was oxidized at 200 °C in 200 mbar of O_2_ for 30 min. For reduction, the sample was exposed to 100 mbar of H_2_ at 540 °C for 30 min. The Pt 4d, Al 2s, O 1s, and Cl 2p core level spectra were measured at a pass energy of 20 eV.

Diffuse Reflectance Infrared Fourier Transform Spectroscopy (DRIFTS) spectra were measured with an INVENIO R spectrometer (from Bruker) using an MCT detector and a Praying Mantis reaction cell (from Harrick Scientific). The spectra were recorded using 128 scans and a resolution of 2 cm^–1^. Typical DRIFTS experiments were performed as follows. The sample cell was first evacuated to <10^–4^ mbar. Subsequently, the sample was exposed to 20 vol % O_2_/Ar and heated to 200 °C at a rate of 3 °C/min and then kept at this temperature for 30 min while continuously measuring the spectra. The cell was then pumped out to 10^–7^ mbar and cooled down to ∼ −140 °C to record DRIFTS spectra of CO as a probe molecule at a gradually increasing pressure. The sample temperature was measured by a thermocouple placed inside a slightly pressed powder sample. (The second thermocouple was connected to the sample holder and in these experiments showed a temperature close to that of the liquid nitrogen used for cooling.) Finally, the cell was pumped out and warmed up to room temperature before the sample was heated in 10 vol % H_2_/Ar to 540 °C and kept at this temperature for 1 h. Finally, we measured low-temperature CO DRIFTS spectra on the reduced sample as described above.

Thermogravimetric analysis coupled with mass spectrometry (TGA-MS) measurements was performed with a TGA 5500 analyzer (from TA Instruments). The sample (10 mg) was first heated in 25 mL/min flow of 20 vol % O_2_/N_2_ from room temperature to 120 °C for 2 h to desorb water. Then the sample was heated to 800 °C at a rate of 2 °C/min. The gas composition was monitored by a quadrupole mass spectrometer (Hiden Analytical).

Raman spectra were measured using an inVia Reflex spectrometer (Renishaw). The spectrometer was operated using a 785 nm laser and was calibrated with a 520.5 cm^–1^ signal from a Si(100) wafer. To minimize laser-induced sample damage, 0.1% of the source power was applied. Spectra were processed using commercial WiRE 5.5 software.

## 3 Results and Discussion

### 3.1 Catalytic Performance

The pristine and plasma-treated Pt catalysts were tested in the PDH reaction using a reaction procedure including *in situ* oxidation (20 vol % O_2_/Ar, 200 °C), then reduction (10 vol % H_2_/Ar, 540 °C), and finally reaction in the C_3_H_8_:H_2_:Ar (8:1:1) mixture at 540 °C. Figure 1a compares propane conversion and selectivity to propene over the pristine Pt/Al_2_O_3_ catalyst and the same catalyst pretreated with either an Ar or H_2_ plasma prior to the oxidation and reduction steps, henceforth denoted as Pt, Pt-AP, and Pt-HP catalysts, respectively. The comparison revealed that the catalyst treated with the H_2_ plasma exhibits significantly higher propane conversion than the untreated catalyst, whereas pretreatment with the Ar plasma had almost no effect on activity. Remarkably, the gain in propane conversion is not accompanied by a loss of propene selectivity. Apparently, the H_2_ plasma pretreatment increases the number of active sites. This effect lasts for at least the experimental TOS used here and also persists at higher reaction temperatures (Figure S2).

**Figure 1:**
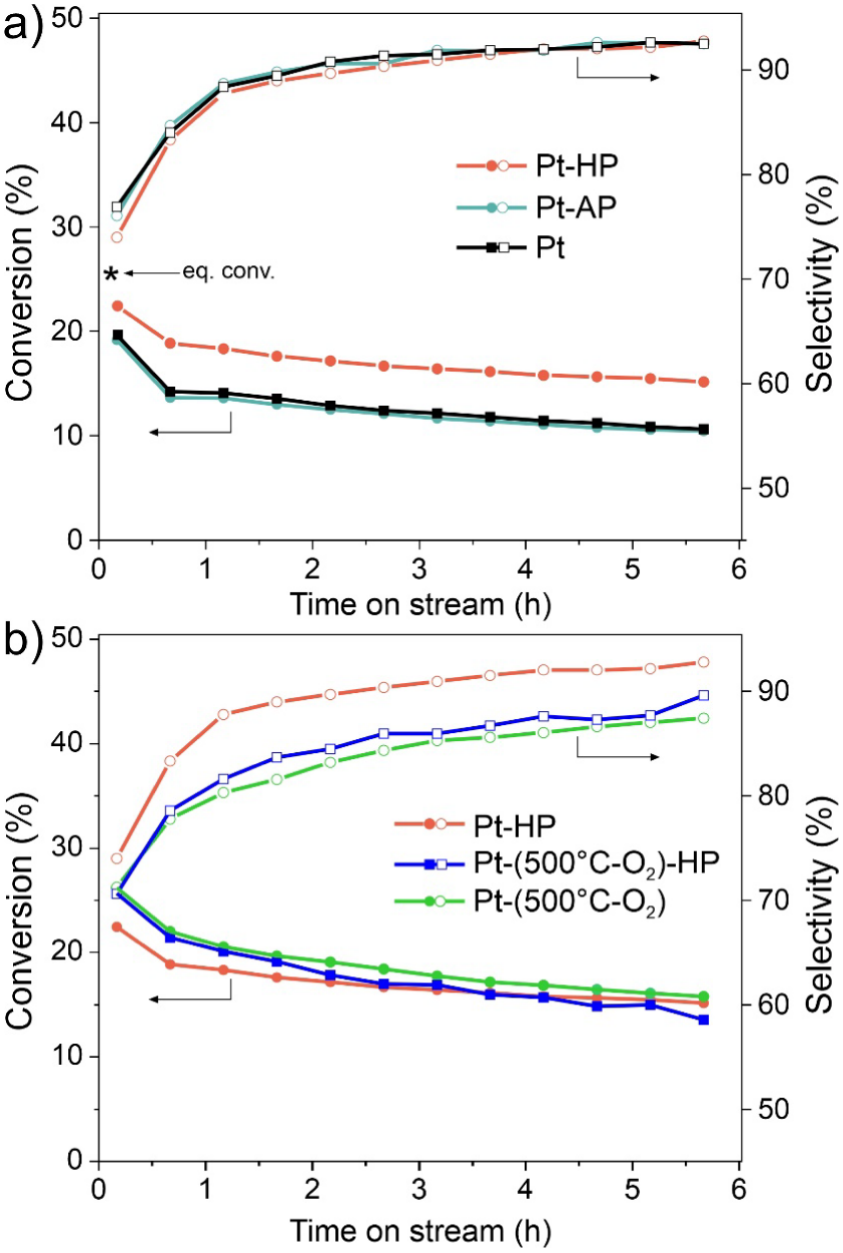
Propane conversion and selectivity to propene measured as a function of time in the C_3_H_8_ + H_2_ + N_2_ (8:1:1) flow at 540 °C. a) Comparison of pristine Pt and H_2_- and Ar-plasma-treated (Pt-HP, Pt-AP) catalysts, which were subsequently calcined in O_2_ at 200 °C and reduced in H_2_ at 540 °C prior to the PDH reaction. The calculated equilibrium propane conversion is indicated by a star. Panel b shows the effect of the H_2_ plasma treatment applied to the Pt catalyst calcined in O_2_ at 500 °C. All samples were reduced in H_2_ at 540 °C prior to the reaction. The results for the Pt-HP catalyst shown in panel a) are reproduced in panel b) for direct comparison.

All catalysts showed deactivation over time, most considerably within the first 30 min. Such kinetics are well documented in the literature for Pt-based catalysts and are usually associated with Pt sintering during the reaction and coke formation.^14–16^ The fact that the selectivity drastically improves during the first hour of TOS implies that it is not sintering but the formation of the active phase that takes place during this period, likely involving a dynamic interchange of several Pt–C structures formed *in situ*.^36^ Deactivation should thus only be considered after the initial performance change, which we identify as being complete after 2 h of TOS. The H_2_ plasma-treated sample showed a lower deactivation rate than the other two catalysts (Figure S3) suggesting that at least in the initial phase of operation, this catalyst exhibits significantly improved stability. Although it is difficult to compare catalytic performances obtained in different studies under different conditions and testing details, our results showed good agreement with the previous findings for Pt/Al_2_O_3_ as well as Pt–Sn/Al_2_O_3_ catalysts prepared mechanochemically^30^ (Table S1).

For comparison, we also examined the H_2_ plasma pretreatment effect on the reactivity of a catalyst that was calcined in oxygen at 500 °C (Figure 1b) to induce Pt clustering before the plasma treatment (Figure S4). Interestingly, the catalyst calcined at 500 °C showed a higher conversion compared to the catalyst calcined at 200 °C, and even slightly higher than the catalyst first treated in the H_2_ plasma and then calcined at 200 °C, although the difference becomes smaller at longer times on stream. However, the high-temperature calcination leads to a substantial decrease in selectivity, and the plasma treatment of the catalyst calcined at 500 °C caused, in essence, no effect on the catalyst’s performance. We can tentatively assign this observation to a difference in the morphology of the Pt particles and the structure sensitivity of the reaction, which shows the trade-off between activity and selectivity of the close-packed and “open” Pt surfaces.^18^ Therefore, the H_2_ plasma showed its beneficial effect on the propene yield only when exposed to the “as-prepared” catalyst before any treatments at high temperatures, i.e., when Pt is in the highest dispersion state.

### 3.2 Catalyst Characterization: Electron Microscopy

The “as-prepared” Pt/Al_2_O_3_ catalysts (i.e., after impregnation and drying) contained only singly dispersed Pt species, as shown in Figure 2a (see more in Figure S5a). Analysis of the HAADF-STEM images showed that the plasma treatment may cause partial clustering of the Pt SACs (Figures 2e and S5a). Nonetheless, after subsequent oxidation at 200 °C and reduction at 540 °C, all catalysts showed Pt NPs of 1.2 ± 0.3 nm in size (Figure 2d,h), although some single atoms could still be found (Figure 2b,f). The images did not reveal discernible differences in the morphology of the untreated and plasma-treated Pt catalysts in the reduced state (Figures S5–S7).

**Figure 2:**
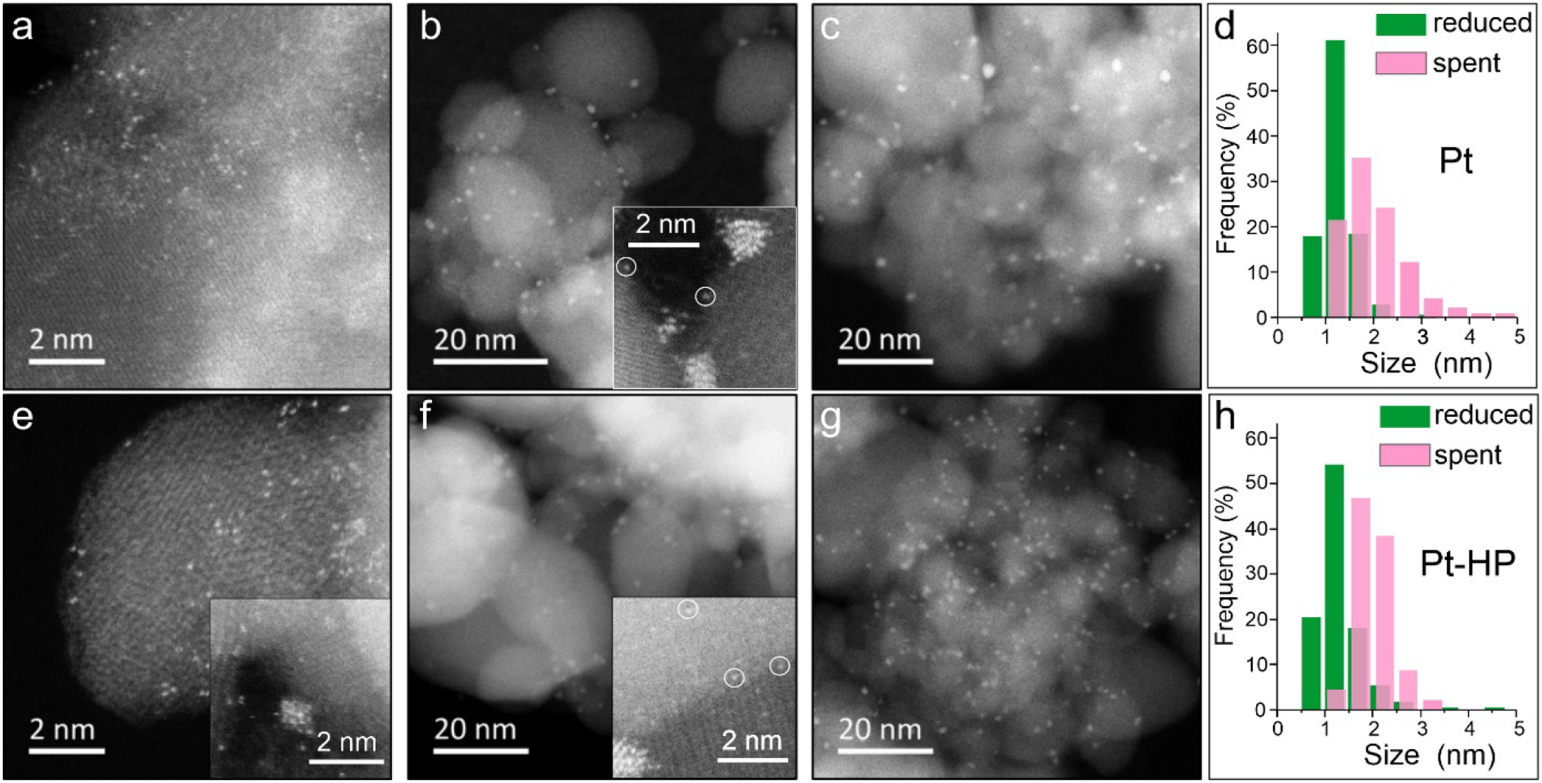
HAADF-STEM images of pristine (a–c) and H_2_-plasma treated (e–g) 0.2 wt % Pt/Al_2_O_3_ catalysts: “as prepared” (a, e); after reduction in H_2_ at 540 °C (b, f), and the PDH reaction at 540 °C (c, g). The Pt particle size distributions of the reduced and postreaction Pt and Pt-HP catalysts are shown in (d) and (h), respectively. The inset in (e) illustrates the Pt particle formation after the H_2_ plasma treatment. The circles in the insets (b, f) mark some Pt single atoms that still remained in the reduced catalysts.

During the PDH reaction, further Pt sintering takes place. STEM images of the catalysts after 6 h of reaction showed considerably larger Pt NPs (∼2 nm in size, on average). Although the size distributions in the Pt and Pt–H_2_ catalysts are slightly different (Figure 2d,h), the corresponding Pt dispersions estimated based on the STEM data were nearly the same and hence cannot account for the ∼30% difference in propane conversion as observed (Figure 1a). Furthermore, direct measurements of the Pt dispersion in these two reduced catalysts using CO uptake yielded very close values (100% and 96% for the Pt and Pt-HP catalysts, respectively), suggesting that the observed difference in reactivity cannot be explained by changes in the Pt surface area.

### 3.3 X-ray Absorption Spectroscopy (XANES and EXAFS)

The electronic state and structure of Pt in the catalysts were investigated by XAS. The XANES spectra at the Pt L_3_ edge and Fourier-transformed (FT)-EXAFS data are displayed in Figure 3a and b, respectively. For the “as-prepared” Pt sample, the XANES spectrum closely resembles that of the H_2_PtCl_6_ precursor. The absence of a Pt–Pt contribution in the corresponding EXAFS spectrum also suggests the presence of isolated Pt species, in full agreement with the TEM results. In contrast, XANES spectra for the plasma-treated samples (both Pt-HP and Pt-AP) exhibited a considerable decrease in the white line intensity, indicating a partial reduction in Pt upon plasma treatment. Fitting the EXAFS spectra further revealed a contribution of Pt–O bonds and weak Pt–Pt bonds (Figures 4b,c, S8–S9 and Table S2) in the plasma-treated catalysts, suggesting that plasma promotes Cl deligation in the precursor and initiates Pt clustering. After calcination in oxygen at 200 °C, both untreated and plasma-treated samples catalysts showed the majority of Pt species in the cationic state, with a strong contribution of the Pt–Cl bonds still being present (Figure S10).

**Figure 3:**
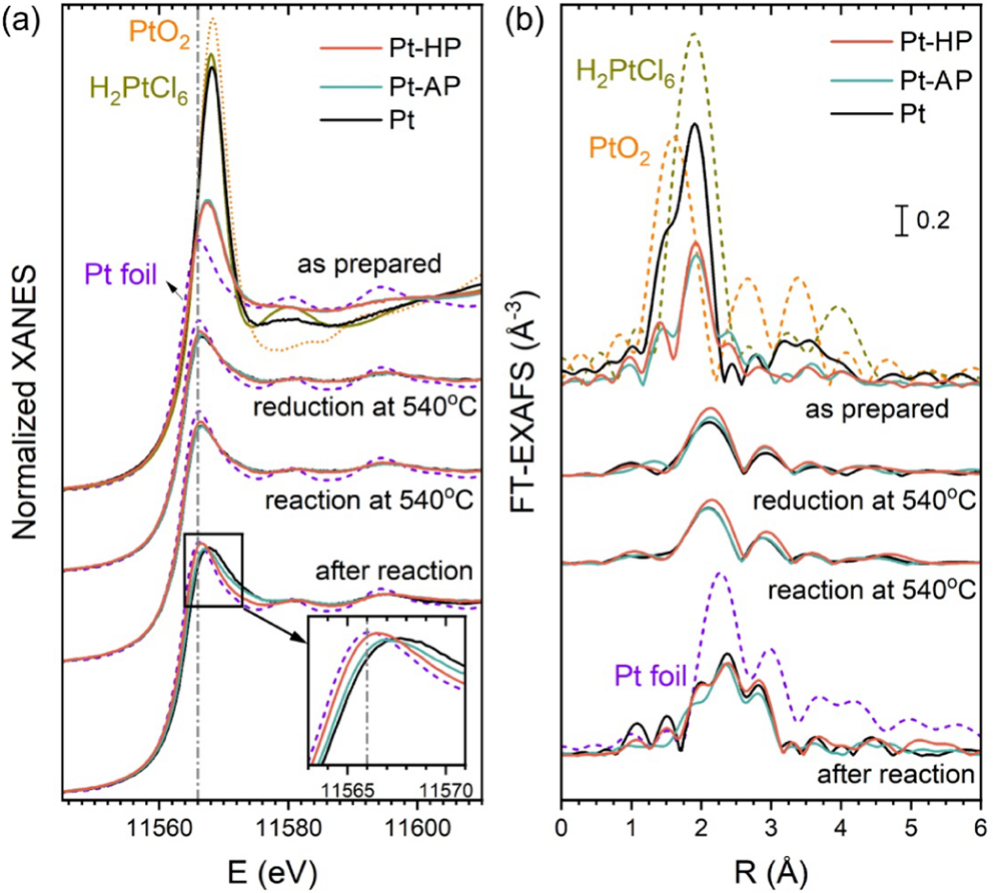
Normalized Pt L_3_-edge XANES (a) and Fourier transforms of *k* ^2^-weighted Pt L_3_-edge EXAFS spectra (b) of untreated (Pt) and plasma-treated (Pt-AP, Pt-HP) catalysts obtained (from top to bottom) on the “as-prepared” samples at room temperature; in H_2_ atmosphere at 540 °C; during the PDH reaction at 540 °C; and at room temperature in He after the reaction. Reference spectra for Pt foil, Pt precursor (H_2_PtCl_6_), and PtO_2_ are shown for comparison (dashed lines). The *k*-ranges used for Fourier transforms are 3–12 and 3–9 Å^–1^ at room temperature and 540 °C, respectively.

**Figure 4:**
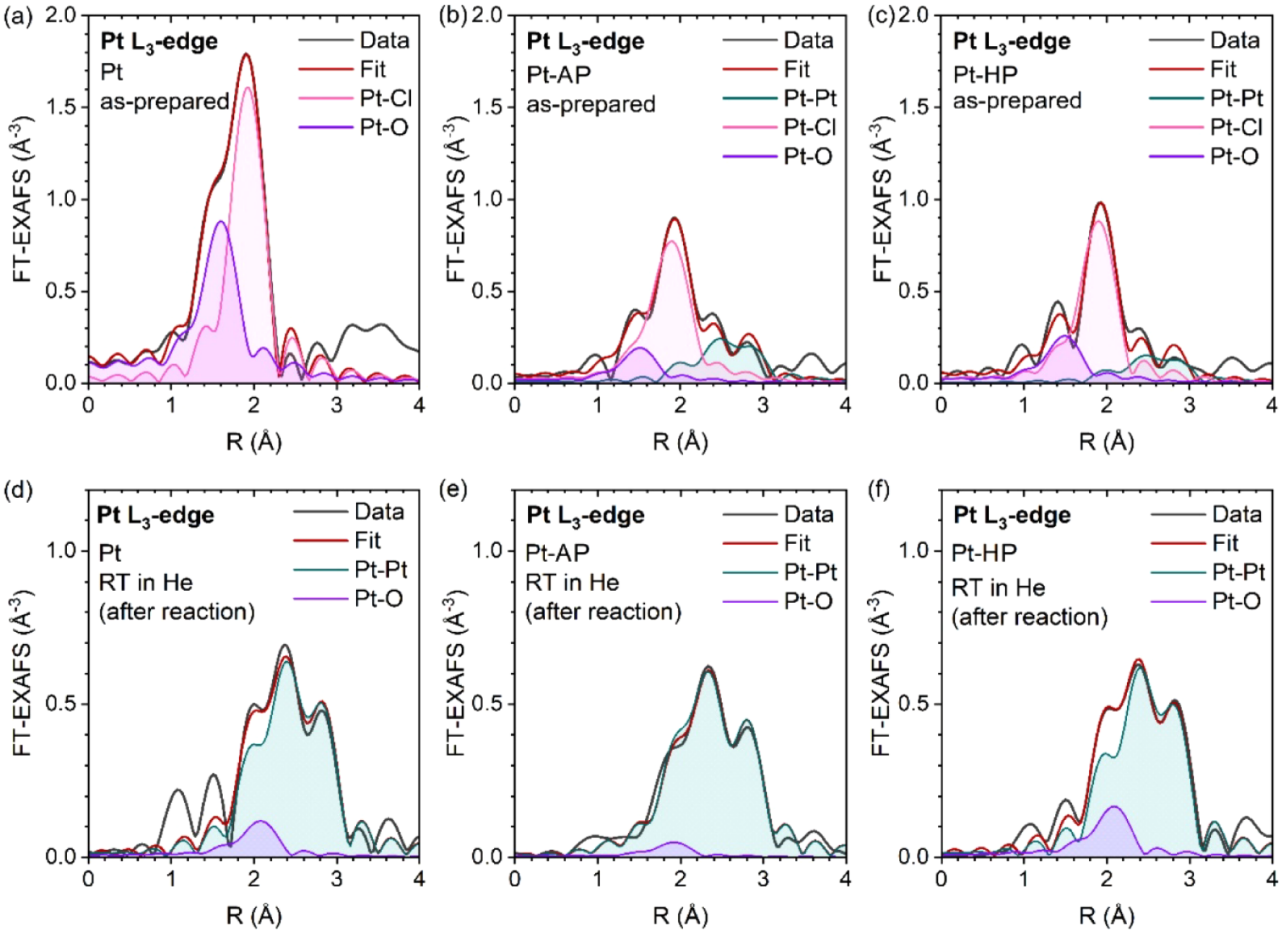
First-shell EXAFS fitting results for the “as-prepared” Pt (a), Pt-AP (b), and Pt-HP (c) catalysts, and the corresponding postreaction Pt (d), Pt-AP (e), and Pt-HP (f) catalysts.

During the subsequent *in situ* reduction in 1 bar of 20 vol % H_2_/He mixture at 540 °C and the PDH reaction at 540 °C, the XANES spectra of all samples become very close to that of metallic Pt. We can only note a slight shift toward higher energy in the white line for Pt and Pt-AP, but not for the Pt-HP samples. The XAS spectra for the reduced samples and the samples under the reaction conditions are virtually identical. Some minor differences become visible when the catalysts were cooled down to room temperature in He after the reaction (inset in Figure 3a). This difference could be partially a result of the effect of the residual H coverage, which is known to affect the shape of the Pt white line.^37–40^ While hydrogen is expected to be fully desorbed at 540 °C,^41,42^ readsorption of H_2_ can occur during the cooling of the catalyst in the presence of residual H_2_ in the gas dosing system. Note also that hydrogen adsorption leads to a better atomic ordering of small Pt NPs.^43,44^

Concomitantly, FT-EXAFS data for reduced samples showed a main peak at ∼2.2 Å (phase-uncorrected) that corresponds to the Pt–Pt bond in Pt NPs and dominates in all spectra of the reduced samples and all samples under reaction conditions (Figures 3b, S8d–i and S9d–i). Considering that the analysis of the EXAFS spectra collected at high temperatures is prone to significant systematic errors, the interpretation of the structural parameters extracted from the high-temperature EXAFS data should be done with caution.^45^ Nonetheless, in agreement with the XANES data, the differences in the EXAFS data collected under the reduction and reaction conditions between Pt, Pt-AP, and Pt-HP catalysts are subtle. Careful analysis of the postreaction data collected at room temperature indicated that, in all samples, the final Pt–Pt coordination number (5.8–8.6) is much lower than that of bulk Pt metal (=12). Furthermore, the Pt-HP sample showed a slightly lower Pt–Pt coordination number than both the Pt and Pt-AP samples (Figure 4d–f and Table S2). In addition, the inclusion of a small contribution from Pt–O bonds with a relatively long length (∼2.5 Å) resulted in a better fit to the experimental EXAFS data collected after cooling the sample to room temperature (Figures S8j–l, S9 j–l and Table S2). Such bonds can reasonably be associated with the interface between the Pt NPs and the alumina support.^38,46,47^ A lower Pt–O and higher Pt–Pt coordination number in the Pt-AP sample could indicate a more three-dimensional character of Pt NPs with a reduced metal–support interfacial area, whereas a higher Pt–O and lower Pt–Pt coordination number in Pt-HP are consistent with a flattened, raft-like structure interacting strongly with the alumina support via the Pt–O bonds. On the other hand, STEM images (Figure 1) showed that the reduced catalysts still contain singly dispersed Pt species, which are obviously coordinated by oxygen in alumina, and these could also contribute to the observation of the Pt–O bonds and the reduced Pt–Pt coordination numbers obtained from the analysis of sample-averaged data. However, based on our XANES results showing that the spent catalysts are predominantly metallic, the contribution of the remaining singly dispersed Pt species is concluded to be rather minor. Thus, the reduced EXAFS amplitude and the reduced Pt–Pt coordination numbers observed for our samples are likely stemming from the small average size of the Pt particles (hence, large surface-to-volume ratios^48^) and in the case of Pt-HP, from the possible formation of raft-like particles. It should be, nonetheless, considered that the accompanying information from microscopy measurements is insufficient to make unambiguous statements about the final structure of the catalyst, considering the complexity of the system and the low spectroscopic contrast between different catalysts. Moreover, the state of the catalyst that we derive from the room temperature TEM or XAS data analysis measurements might be different from the active state of the catalyst that is only present during reaction at the elevated reaction temperature and that includes carbon atom incorporation into the Pt NPs.^36^

### 3.4 X-ray Photoelectron Spectroscopy (XPS)

Now we address the surface chemical composition and electronic state of Pt in our catalysts as studied by XPS. In order to avoid adventitious surface contamination during the sample transfer from the plasma and catalytic reactors to the XPS chamber, we have made use of a vacuum-compatible plasma source in the UHV chamber to simulate the catalyst treatment in the plasma setup. In addition, we used a high-pressure reaction cell attached to the analytical chamber for the oxidation and reduction steps at near-atmospheric pressures. As the most intense Pt 4f signals overlap with the Al 2p peak of alumina, the electronic state of Pt was monitored based on the Pt 4d core level (namely, the Pt 4d_5/2_ line). In addition, we also examined the Cl 2p region since the reactivity of Pt group metal catalysts prepared using chlorinated precursors, such as H_2_PtCl_6_ in our case, is found to be quite sensitive to Cl residues (see refs 49,50 and references therein).

Figure 5a displays quasi *in situ* Pt 4d and Cl 2p spectra measured first on the “as-prepared” catalyst, then after *in situ* treatment with H_2_ plasma (at 10^–4^ mbar), after 0.2 bar O_2_ at 200 °C, and finally after 0.1 bar of H_2_ at 540 °C, each for 30 min. For comparison, the results of the oxidation–reduction treatments on the untreated (Pt) catalyst are shown in Figure 5b. Note that due to the low Pt loading in our catalysts and the relatively low sensitivity factor of the Pt 4d lines, the spectra had to be recorded with acquisition times of about 7 h each in order to improve the otherwise relatively low signal-to-noise ratio.

**Figure 5:**
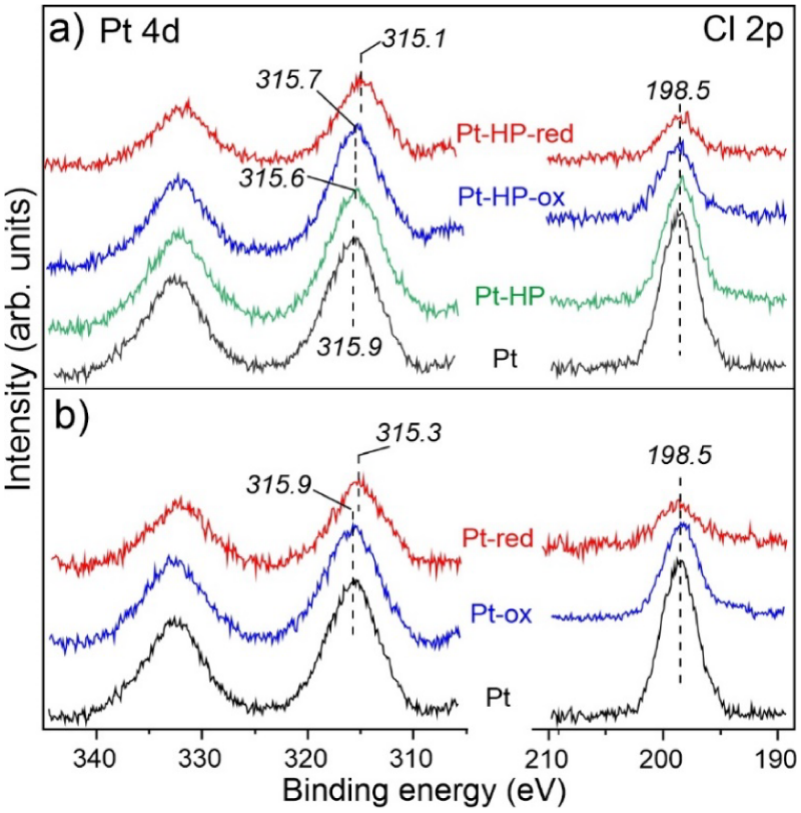
(a) Quasi *in situ* Pt 4d and Cl 2p XPS spectra measured on the “as-prepared” Pt/Al_2_O_3_ catalyst (Pt); after low-pressure H_2_ plasma treatment (Pt-HP); after subsequent oxidation in 0.2 bar of O_2_ at 200 °C (Pt-HP-ox); and after reduction in 0.1 bar of H_2_ at 540 °C (Pt-HP-red). The results of the same experiments on the untreated (Pt) sample are shown in panel (b). The BEs were calibrated, and the signal intensities were normalized using the Al 2s peak set to 119.0 eV. The BE values shown for the Pt 4d_5/2_ signals are determined by CasaXPS using a single peak fitting.

Nonetheless, the BE of the Pt 4d_5/2_ state (315.9 eV) obtained for the “as-prepared” Pt SAC catalyst corresponds to Pt in the oxidation state 2+. The BE of the Cl 2p state (centered at around 198.5 eV, spin–orbit unresolved) is typical for metal chlorides.^51^ Based on the atomic sensitivity factors of Pt 4d and Cl 2p lines (3.5 and 0.89, respectively^50^), the Cl:Pt atomic ratio in the pristine sample is about 2.5, i.e., much smaller than 6 in the stoichiometric H_2_PtCl_6_ precursor, consistent with the generally accepted ligand exchange mechanism of anchoring the Pt precursor onto the hydroxylated alumina^52^ surface during impregnation.

The H_2_ plasma considerably reduces the amount of Cl at the surface and also shifts the Pt 4d lines to lower BEs (315.6 eV), indicating a partial reduction of the Pt^2+^ species to Pt^0^. Direct comparison of the untreated and HP-treated samples showed a weak signal from the low BE side (see the difference spectrum in Figure S11). The latter can be assigned to partially reduced Pt species forming the Pt–Pt bonds as shown by XAS. Subsequent oxidation at 200 °C slightly (by +0.1 eV) shifts the Pt 4d lines. However, the oxidized state of Pt dominates on both plasma-treated and untreated samples. The amount of Cl decreases upon oxidation at 200 °C. Exposure to 100 mbar of H_2_ at 540 °C reduces the Cl content considerably, almost to a negligible level. A strong shift of the Pt 4d_5/2_ peak to the lower BEs and its considerably reduced intensity are fully consistent with the formation of metallic Pt NPs upon reduction in H_2_ as shown by TEM and XAS. There is a small difference between the BE values obtained for the untreated and plasma-treated samples in the reduced state, i.e., 315.3 vs 315.1 eV, respectively. In principle, this may arise from (i) different sizes of the Pt NPs formed upon reduction; (ii) different contributions of the Pt–O bonds at the metal/support interface; and (iii) a combination of both. It is well-known that the binding energy measured on metal NPs experiences a considerable shift toward higher BEs for the smallest particles, which often overlaps with that of partially oxidized species. This renders the accurate spectral deconvolution of the Pt signals difficult.

All in all, the XPS results show that the catalyst pretreatment with the H_2_ plasma substantially decreases the amount of Cl at the surface. We should recall, however, that the UHV-compatible plasma source used here operates at a much lower pressure as compared to that used for the reactivity studies (10^–4^ mbar vs ∼5 mbar). Accordingly, the H_2_ plasma at near-atmospheric pressures is expected to reduce the Cl content to a much larger extent. Although the oxidation at 200 °C reduces the Cl concentration in both untreated and plasma-treated samples, only the high-temperature treatment in H_2_ can largely eliminate Cl. However, the amount of Cl residues in these two catalysts as revealed by XPS is essentially the same. Therefore, the observed plasma effects on Cl alone cannot explain its effect on the catalytic performance.

We also analyzed the Al 2s and O 1s spectra to detect possible chemical modifications of the alumina support surface after different treatments. The results (Figure S12) showed a small but detectable broadening of both the Al 2s and O 1s lines after the H_2_ plasma treatment, which can be assigned to an enhanced structural disorder of the alumina surface.^53^ However, the difference vanishes after the first annealing at 200 °C in O_2_.

The combined XPS and XAS results allow us to propose that the plasma treatment interferes with the formation process of the Pt nanoparticles and creates differently functioning nanoparticles that are not lost during subsequent high-temperature reactions. Following our perception, we can assume that the hydrogen plasma treatment creates low-coordinated Al sites and oxygen vacancies. The plasma-assisted dechlorination in the Pt precursor is thought to be beneficial for the creation of a stable functional interface between Pt (nuclei) and the activated alumina surface.

### 3.5 Diffuse Reflectance Infrared Fourier Transform Spectroscopy (DRIFTS)

To shed more light on the surface structure of the Pt catalysts, we employed DRIFTS, using CO as a probe molecule to characterize the state of Pt and the alumina support during various preparation treatments. Figure 6a displays *in situ* DRIFTS spectra obtained for the untreated Pt catalyst during calcination in 20 vol % O_2_/Ar at 1 bar. First, we note that the “as-prepared” catalysts (i.e., dried in vacuum at 60 °C overnight) show a substantial degree of surface hydroxylation, manifested by a strong and very broad ν­(OH) band centered at ∼3400 cm^–1^ and originating from hydrogen-bonded hydroxyl species. This band dominates the initial spectra and therefore makes it difficult to identify the high-frequency bands related to isolated hydroxyls. Nonetheless, the initial spectrum featuring bands at around 3780, 3740, 3720, and 3680 cm^–1^ and its evolution on heating show certain similarities to those previously reported for pristine α-Al_2_O_3_ samples synthesized by ball milling and subsequently calcined in air at elevated temperatures.^29^ The spectra are also consistent with those reported for “conventional” α-Al_2_O_3_ (partially dehydroxylated via heating to 500 °C), showing principal bands at 3742 and 3704 cm^–1^.^54^

**Figure 6:**
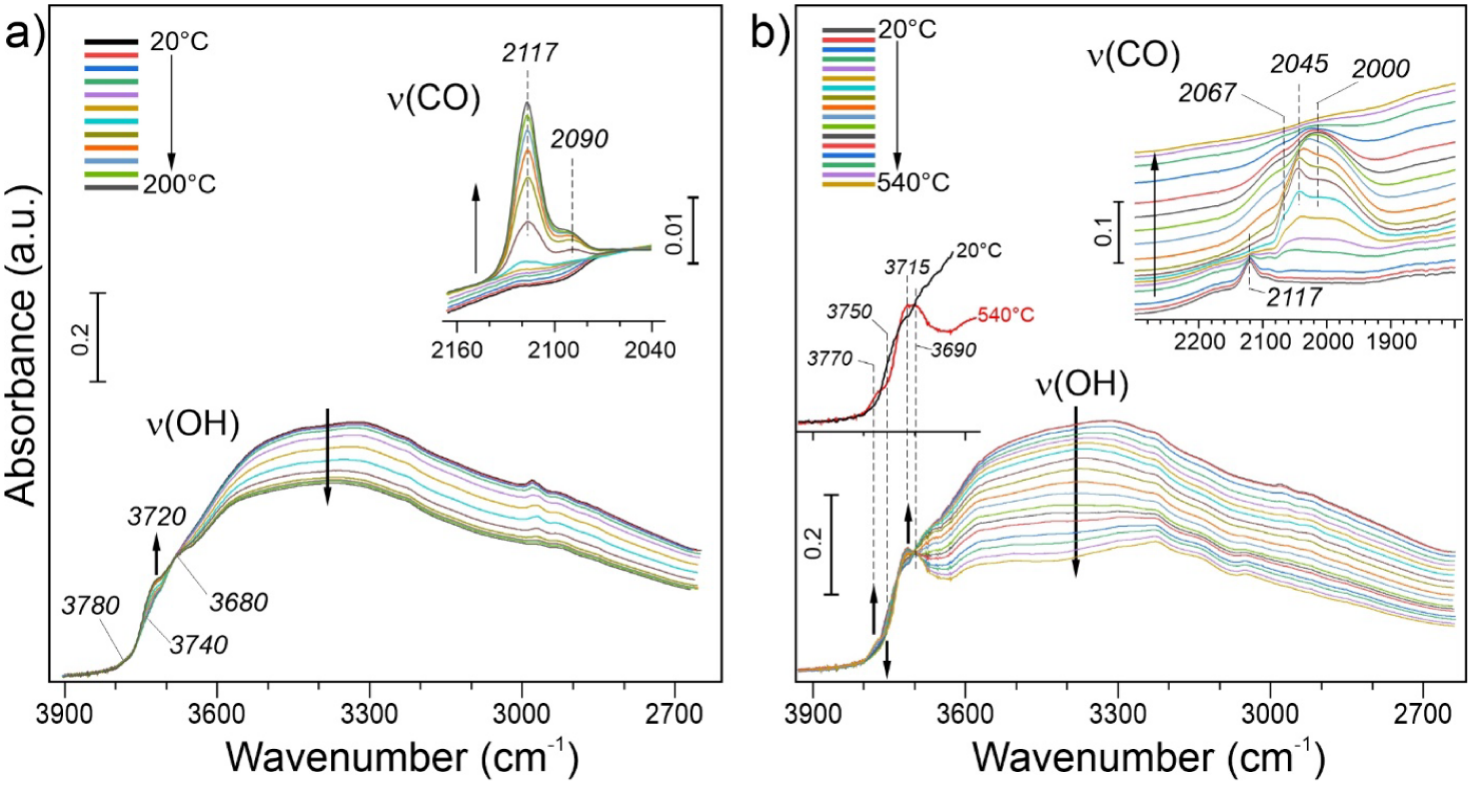
(a, b) The ν­(OH) region and ν­(CO) region (insets) in *in situ* DRIFTS spectra measured on “as-prepared” Pt/Al_2_O_3_ catalyst during heating from room temperature to 200 °C in O_2_/Ar (a), then cooled down to room temperature and heated to 540 °C in H_2_/Ar (b). In panel (b), the first (i.e., at 20 °C) and the last (at 540 °C) spectra are reproduced for direct comparison. (The multiple bands at ∼2950 cm^–1^ are assigned to ν­(CH_*x*_) vibrations in adventitious carbonaceous species.).

During heating in O_2_, the catalyst undergoes typical dehydration/dehydroxylation via water desorption, which is also accompanied by redistribution of the remaining hydroxyl species. As a result, the band at ∼3720 cm^–1^ gains in intensity, and the band at ∼3680 cm^–1^ becomes more prominent, as shown in Figure 6a. More striking, however, is the appearance of a sharp band at 2117 cm^–1^ and a much weaker band at 2090 cm^–1^ in the ν­(CO) region upon heating to temperatures above 170 °C (see inset in Figure 6a), which were never observed on the pure alumina samples. Both bands show no shift with increasing signal intensity, thus indicating that CO species are adsorbing on isolated sites. The bands fall in the range previously reported for several Pt SACs^55–57^ and can therefore be assigned to CO bonded to Pt single atoms in our catalysts. The fact that the ν­(CO) bands appear presumably from traces of CO in the flow of O_2_ points to a high sticking coefficient of CO to these sites. Subsequent exposure to CO up to 60 mbar at −135 °C showed additional CO adsorption bands on alumina (see more below) but almost no changes in the CO/Pt region (Figure S13a), indicating that CO saturated all the Pt sites available during heating in O_2_ above 170 °C.

It should be noted, however, that the precise assignment of the IR bands based solely on their position is not straightforward and remains debated in the literature^58–60^ (see Table S4). For example, the 2120 cm^–1^ band has previously been reported for both calcined and reduced Pt/Al_2_O_3_ catalysts at relatively high metal loadings (1–4 wt %) and assigned to strong CO adsorption on oxidized Pt sites.^61,62^ A similar band was reported for Pt catalysts supported on zeolite HZSM-5 and SiO_2_ at various loadings (0.5–2.5 wt %) and assigned to CO adsorbed on Pt single atoms.^56^ Importantly, the band was observed in the DRIFTS spectra irrespective of the Pt precursor used in the studies mentioned above (i.e., MeCpPtMe_3_, Pt­(NH_3_)_4_(NO_3_)_2_, or H_2_PtCl_6_). Moreover, the band remained in the O_2_-rich CO + O_2_ atmosphere at elevated temperatures (∼175 °C), pointing to strong CO bonding to these Pt sites and to the fact that O_2_ cannot dissociate on Pt species to react with CO that adsorbs strongly. In a similar manner, we can also assign the 2120 cm^–1^ band observed on our catalysts to strongly adsorbed CO on the cationic Pt SAs.

The same type of experiments on Ar and H_2_ plasma-treated catalysts revealed similar behavior to that of the untreated catalyst. All three catalysts showed the bands at around 2120 and 2090 cm^–1^ on heating in O_2_ to 200 °C, although their intensity ratio may deviate from sample to sample (Figures S13a–c andS14).

*In situ* DRIFTS spectra recorded during subsequent reduction in 10 vol % H_2_/Ar are displayed in Figure 6b. We first note further dehydroxylation of the alumina surface on heating to 540 °C, which is accompanied by the formation and/or redistribution of isolated hydroxyl species. For direct comparison, the first and last spectra are reproduced in the inset of Figure 6b. The band appearing at ∼3770 cm^–1^ at the expense of the band at 3750 cm^–1^ is typically associated with terminal OH on the low-coordinated Al sites. Although precise assignment is still debated in the literature^54^ (Table S3), it appears that hydrogen, indeed, creates defects in the oxygen sublattice, allowing low-coordinated Al species to become frequent at the surface. The critical role of under-coordinated Al sites for strong bonding of Pt species was reported in a prior study^63^ targeting the penta-coordinated Al sites. Both observation by microscopy and theory support the concept that defect formation on the α-Al_2_O_3_ surface at the state of the molecular Pt precursor should be a potent method for the creation of a stable functional interface of metallic Pt.

In the ν­(CO) region (inset in Figure 6b), the bands at 2090–2120 cm^–1^ attenuate upon the introduction of H_2_ at room temperature and ultimately disappear on heating above 130 °C. Concomitantly, new bands appear in the 2070–2000 cm^–1^ region, which fall within the range of frequencies obtained for CO on Pt NPs and thus can be associated with intermediate Pt metal aggregates that form from the Pt SAs upon heating in H_2_. Note also that the ν­(CO) frequencies may be affected by coadsorbed H atoms on the Pt surface. The CO bands disappear above 500 °C but reappear during sample cooling and pumping H_2_ out, due to the well-known high sticking coefficient for residual CO on the Pt surfaces.

Figure 7a compares the CO DRIFTS spectra obtained for the reduced Pt, Pt-AP, and Pt-HP catalysts, first in 55 mbar of CO and then in a vacuum, all at ∼ −135 °C. The spectra feature a main peak at around 2065 cm^–1^, with a long tail extending to 1900 cm^–1^. This type of spectra are well documented for Pt NPs and commonly assigned to a linear CO adsorption on particle terrace sites and on particle corners/kinks sites, respectively^64,65^ (see Table S4). Also, the position of the main band and its blueshift with increasing CO pressure (Figure S13d–f) are typical for Pt NPs.^56,64–66^ In fact, the spectra for reduced Pt, Pt–AP, and Pt–HP catalysts look similar at first glance. It should be mentioned here that Cl residues, if present, mostly affect bridge-bonded CO on the particle edges, which are reflected by a broad band centered at ∼1800 cm^–1^.^49^ Comparative analysis of the CO DRIFTS spectra on untreated and plasma-treated catalysts in the reduced state, as presented in Figure S13d–f did not show such an effect. Therefore, we can exclude Cl residues as a potential explanation for the beneficial effect of hydrogen plasma. Again, this is n line with XAS and XPS characterization results.

**Figure 7:**
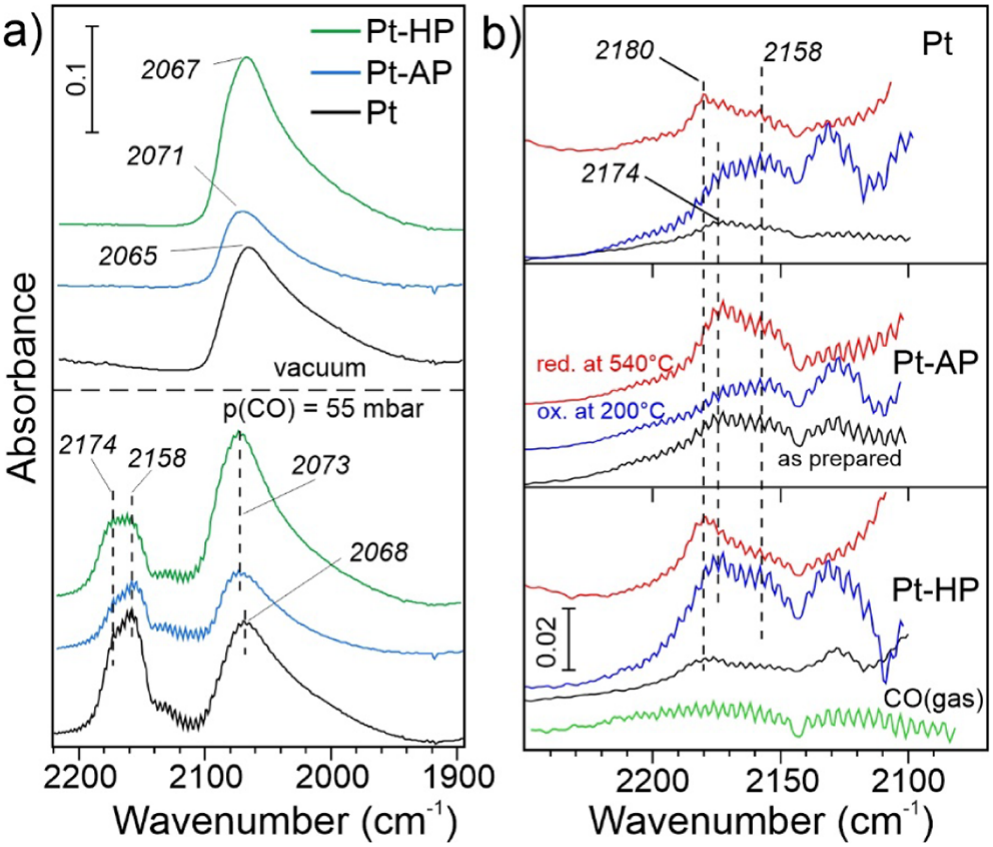
(a) CO DRIFTS spectra for Pt, Pt-AP, and Pt-HP catalysts after reduction in H_2_/Ar at 540 °C. The spectra were measured in 55 mbar of CO and then in vacuum, all at ∼ −135 °C. The spectra are referenced to the baseline measured on the same samples in the “as-prepared” state in the Ar flow at room temperature. (b) Comparison of low-temperature CO DRIFTS spectra highlights the initial stages of CO adsorption on alumina at −135 °C (at 10 mbar) for the “as-prepared” state and after oxidation at 200 °C and reduction at 540 °C; see the color code in the middle panel. The spectra are referenced to the corresponding spectrum measured in vacuum before CO dosing. The band at around 2130 cm^–1^ originated from the CO adsorbed on Pt SAs in the oxidized samples. The bands overlap with two rotational branches of the ν­(CO) band of CO in the gas phase, which are also shown.

We further took a closer look into the plasma-induced modification and evolution of the alumina support. In principle, one could anticipate considerable morphological changes to the alumina surface upon bombardment by “heavy” Ar^+^ species^53^ compared to much lighter species in the H_2_ plasma, which, in turn, can form OH species on the oxide surface. The fact that only the H_2_ plasma pretreatment affected the PDH reactivity suggests that the observed effect lies in the formation process of the active phase and not in a persistent structural difference in the support surface morphology. To investigate the state of alumina using CO adsorption, we analyzed the ν­(CO) bands in the 2140–2200 cm^–1^ region, i.e., typical for alumina surfaces and only develop in the low-temperature DRIFTS spectra at elevated CO pressures, disappearing upon CO pumping (Figure 7a) due to the very weak CO bonding to Al_2_O_3_.^67,68^ Besides the rotational branches of the ν­(CO) band in the gas phase, the low-frequency CO/Al_2_O_3_ region for the oxidized catalysts is additionally obscured by the band at 2120 cm^–1^ associated with CO on Pt SAs. Nonetheless, it is clear that the band at 2174 cm^–1^ appears first, before the band at 2158 cm^–1^ starts to dominate at elevated CO pressures (see the full set of spectra in Figure S13). Although absolute frequencies may depend on the alumina polymorph,^54,68,69^ the high-frequency band is commonly associated with terminal CO on Lewis acid sites (Al^3+^). In general (see Table S4), the higher the frequency, the lower the coordination of the Al sites.^54^ The low-frequency band is mostly attributed to more weakly bound CO on surface hydroxyls,^67^ which are quite abundant in our samples (see Figure 6).

Figure 7b displays selected spectra to illustrate the initial stage of CO adsorption on the alumina support for three different catalysts (Pt, Pt-AP, and Pt-HP) as prepared and after subsequent *in situ* oxidation and reduction. Basically, no spectral differences are found between the Pt and Pt-AP samples in both the as-prepared and oxidized states. However, the “as prepared” Pt-HP sample showed the band at 2180 cm^–1^, which is blue-shifted as compared to the Pt and Pt-AP catalysts, indicating a stronger undercoordination of the Al sites. However, such a band at 2180 cm^–1^ is also observed on the Pt catalyst after reduction in H_2_ at 540 °C, whereas no such feature is detected on the Pt-AP catalyst. Therefore, we can conclude that the H_2_ plasma at near room temperature causes an effect similar to that of molecular H_2_ at high temperature, both creating additional highly undercoordinated Al^3+^ sites, which affect the aggregation of the Pt SAs during the subsequent calcination and reduction steps. In contrast , the Ar plasma seems to remove such Al sites, presumably via a sputtering effect.^53^

To gain more insights into H_2_ plasma-induced surface modification of alumina, we carried out DRIFTS measurements on the sample treated with D_2_ plasma to spectroscopically discriminate plasma-induced species. The spectrum measured at room temperature after the D_2_ plasma treatment (Figure 8) revealed a broad ν­(OD) signal in the 2700–2200 cm^–1^ region in addition to the initially present ν­(OH) bands. The blank experiment, involving exposure to molecular D_2_ without plasma ignition, did not reveal ODs, in agreement with previous results.^70,71^ Therefore, the formation of OD species on the plasma-treated samples can be attributed to two reactions: (i) the H–D exchange reaction between D^+^ ions in the plasma and surface OH hydroxyls; (ii) the reaction of D^+^ with O^2–^ ions in Al–O–Al entities, particularly if the latter are in a strained configuration that facilitates the breaking of the Al–O bonds.

**Figure 8:**
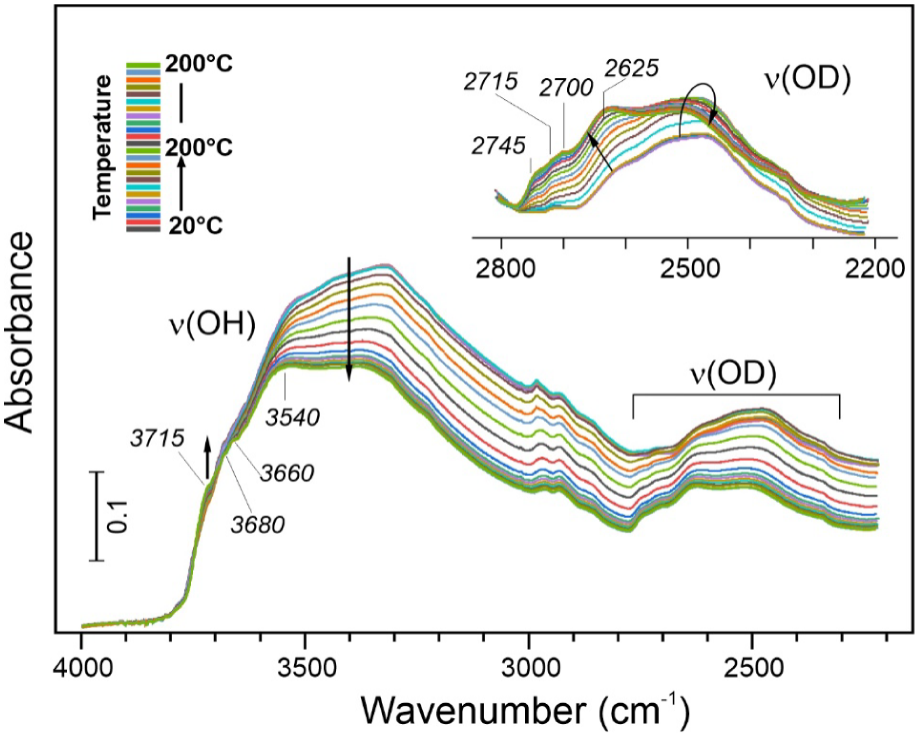
*In situ* DRIFTS spectra of the Pt/Al_2_O_3_ catalyst pretreated with a D_2_ plasma, measured during sample heating in O_2_ to 200 °C. The ν­(OD) region is replotted in the inset, with the spectra aligned to the baseline at the higher frequencies for direct comparison.

Figure 8 displays the spectral evolution of the ν­(OH) and ν­(OD) bands during subsequent heating to 200 °C in oxygen. As expected, the alumina surface undergoes dehydration/dehydroxylation. Remarkably, this process primarily involves OH and not OD species. In fact, the integral intensity of the ν­(OD) signal is even slightly increased (inset in Figure 8), with the new bands appearing at 2745, 2715, 2700, and 2625 cm^–1^. Using the scaling factor 1.356 for the ν­(OH)/ν­(OD) ratio,^71,72^ these bands correspond to 3722, 3682, 3662, and 3560 cm^–1^, respectively, for the ν­(OH) counterparts, which match well with the bands observed in the ν­(OH) region, thus suggesting their identical nature. Tentatively, we can explain the formation of additional ODs by the thermally induced migration of D^+^ species, “implanted” in the alumina bulk during plasma treatment, to the surface. Nonetheless, the ν­(OD) signal attenuates in the H_2_/Ar flow at atmospheric pressure and fully disappears at 540 °C (not shown here), via either the recombinative desorption through D_2_O water formation and/or the reverse D–H exchange reaction between H_2_ in the gas phase and surface ODs. A schematic illustration of the surface modification of Al_2_O_3_ is depicted in Figure 9.

**Figure 9:**
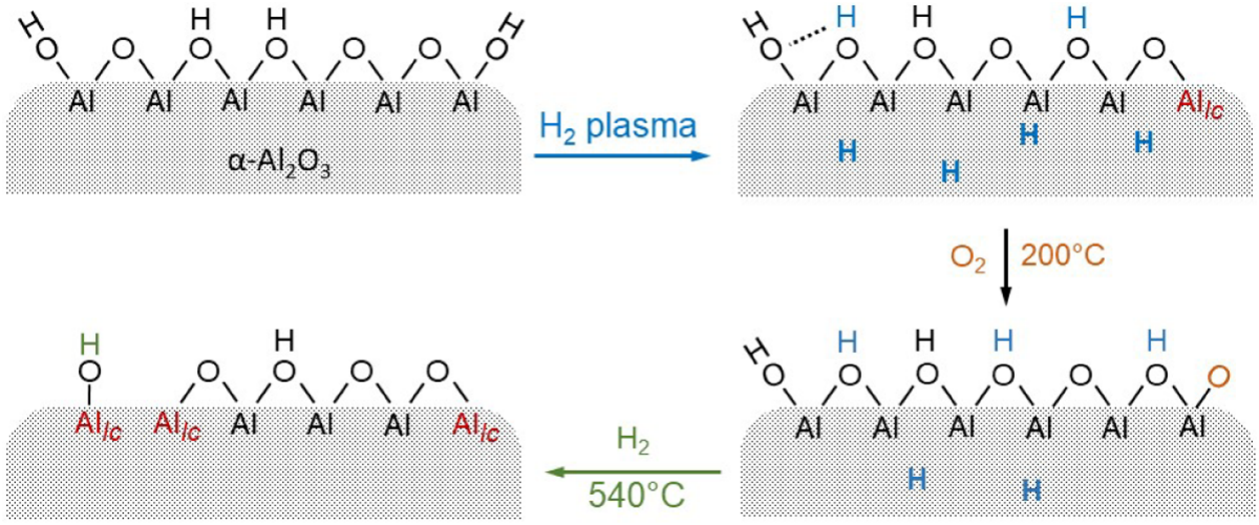
Schematic presentation of surface modification of alumina support by hydrogen plasma and subsequent oxidation and reduction steps, as deduced from the DRIFTS results. (Al_*lc*_ denotes the low-coordinated Al atoms.)

Overall, the DRIFTS results provided additional strong evidence for the formation of Pt NPs upon reduction in H_2_ for all catalysts studied. However, the results showed, in agreement with the other characterization data, that the plasma effect seen in the catalytic data leaves only hard-to-detect differences in the nature of the Pt species. As the Pt loading is small and the difference in electronic structure between the Pt nanostructures of the three catalysts is also small, the distinction is at the limit of what the data can resolve. Nonetheless, if we compare the low-temperature CO DRIFTS spectra for the reduced forms of the three catalysts shown in Figure 7a, we observe a considerably higher intensity ratio of the CO/Pt and CO/Al_2_O_3_ related bands for the Pt-HP catalyst, suggesting the H_2_ plasma-treated sample is the catalyst with the most metal nanostructures. Since the metal loadings in these three catalysts are identical, an increased Pt surface coverage can be achieved by flattening of the Pt NPs into a more two-dimensional particle morphology due to increased adhesion of Pt to the defective alumina surface, in agreement with our EXAFS data. In principle, this fact may explain the enhanced production rate of propene reported in Figure 1.

In all experiments presented above, we observe distributions of characteristic properties and, hence, have to assume that we generated distributions of species rather than a clear selection between Pt SA and nanoparticles with uniform surface and morphology properties. This conclusion leaves room for further systematic improvement of the performance of this class of Pt PDH catalysts. We recall that the use of the mechanochemically produced high-surface-area α-alumina support seems to be a prerequisite for achieving the homogeneous SAC precursor, which is key to our concept of plasma-mediated nucleation and growth of Pt raft-like particles from singly dispersed Pt species.

### 3.6 The Role of Carbon Species

Carbon has a dual role in the PDH reaction, acting both as poisonous coke requiring regeneration^31,73^ and as a modifier^36^ of the initially metallic Pt catalyst. The balance between these antagonistic functions is determined by the morphology of Pt and its interaction with the support. Large, isotropic, and weakly interacting particles tend to generate much graphitic carbon through dissolution–segregation processes. Small rough and strained Pt nanostructures firmly attached to their support tend to produce hydrogen-rich polymeric carbon (“white coke”) resulting from overdehydrogenation and hydrogenolysis. We note that this function is analogous to the control of selective hydrogenation recently exemplified with laterally condensed Pd catalysts in acetylene semihydrogenation.^74^ Ideally, one needs to create a state of Pt that dissolves a small amount of carbon to moderate overdehydrogenation without storing sufficient atoms to segregate them as graphene.^32^ ^,^ With this concept in mind, we studied the character of deactivating carbon in order to characterize the stability of the Pt NPs. The enhanced stability of the H_2_ plasma-treated catalysts, which is manifested by a lower deactivation rate compared to the untreated Pt catalyst (Figure S3), is a first indication that the Pt NPs are, indeed, different in their character when such plasma treatment is applied. Analysis of the particle size distribution by TEM (Figure 2) revealed an increase in the mean particle size from 1.3 to 1.8 nm during the first 6 h of the reaction. The comparison of the postreaction samples also showed a relatively broader size distribution of Pt in these catalysts, particularly the presence of a higher fraction of larger NPs.

It should be noted, however, that the TEM data were not acquired *in situ* at the high reaction temperature but *ex situ* at room temperature, and thus, the real coverage of carbon on the sample during the reaction, which we hypothesize might also experience dynamic changes, is unknown. Indeed, a recent *operando* HRTEM study^36^ of unsupported, relatively large Pt NPs (∼10 nm, on average) under PDH reaction conditions revealed the temperature-dependent formation of ordered Pt–C polymorphs that correlated with a change in propene selectivity. The analysis also revealed the coexistence of multiple phases in individual NPs, leading to local strain and frustrated phase transitions between the different Pt–C structures. Obviously, such a dynamic picture can hardly, if not impossibly, be captured in our analysis of the postreaction samples, which may undergo phase separation and segregation.

Moreover, TEM inspection of all spent catalysts revealed carbon layers that cover both the Pt particles and the alumina support (Figure 10a). Indeed, the fresh, reduced catalyst, initially white in color turns gray during the reaction, suggesting that most of the carbon deposits actually cover the alumina surface. Since pure alumina did not show the coke formation under the reaction conditions studied, we can conclude that it is the Pt that produces carbonaceous species, which spill over onto the alumina support and form the coke covering the catalyst surface.

TGA-MS analysis of the spent Pt catalyst (i.e., plasma untreated) revealed a weight loss of 0.7%, and its first derivative (d*W*/d*T*) peaked at 400 °C, which correlates with the CO_2_ desorption signal (Figure 10b). Remarkably, the spent Pt–HP catalyst showed almost a 2-fold increase in the total amount of coke (1.3% vs 0.7%), which is also reflected in a proportionally increased CO_2_ (44 amu) signal intensity. The combustion temperature (∼400 °C) is far too low to account for pure graphitic carbon (like soot and carbon nanotubes) that usually burns at about 200 °C higher,^75,76^ and instead points to a coke with substantial hydrogen content, well in agreement with “white coke”. In addition, high-resolution TEM images also revealed graphene-like layers (some marked in Figure 10a). The latter is further manifested in Raman spectra (Figure 10c) showing a sharp G band (at around 1600 cm^–1^) and a much broader D band (centered at 1300 cm^–1^), where the G band represents the ideal graphite lattice vibrations with sp^2^-coordinated C atoms, and the D band reflects disordered (defective) carbon lattices including also C atoms in the sp^3^-coordination. The broad D band is usually deconvoluted into several components denoted as D1–D4.^77,78^ The D1 band is assigned to disordered graphitic lattice, the D3 band to amorphous carbon, and the D4 band to disordered graphitic lattice and polyenes, as well as to a C–H bond deformation mode in the hydrogenated coke. Therefore, a relatively high contribution of the D4 component in the spent catalysts suggests a high fraction of hydrogenated coke, which is slightly higher for the H_2_ plasma-treated sample.

**Figure 10:**
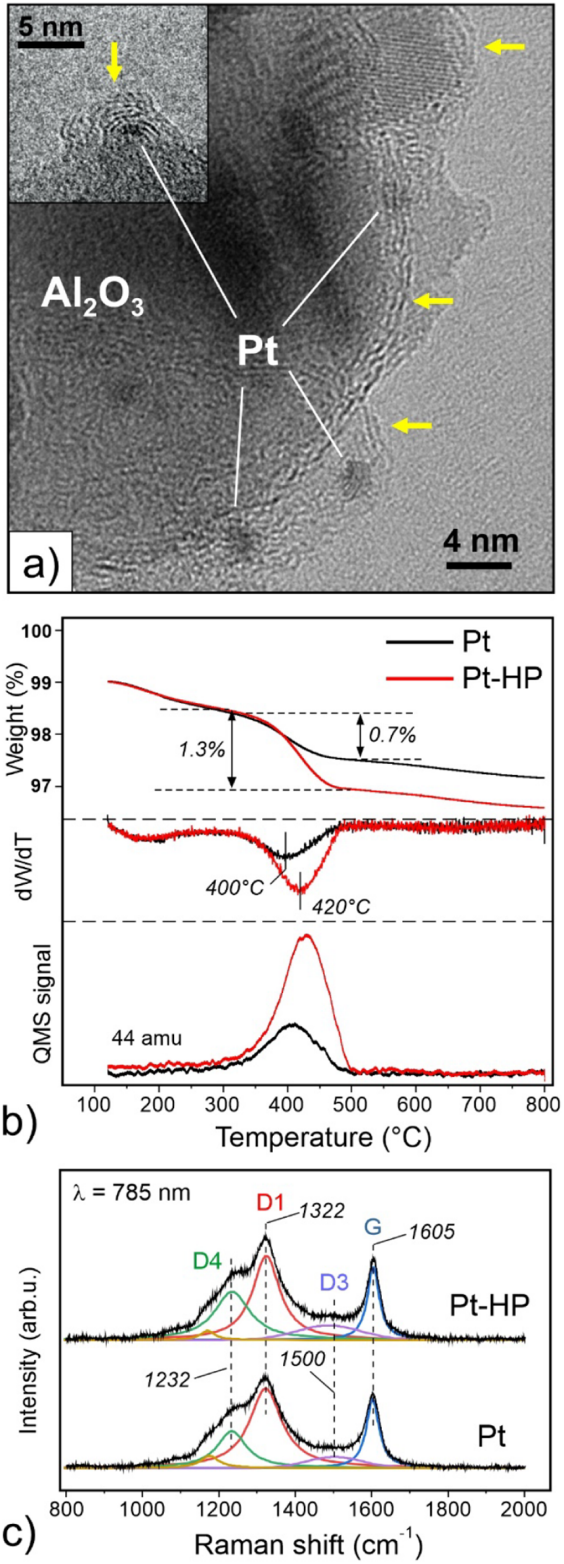
a) Typical bright-field STEM image of the postreaction samples. Carbon layers covering both the Pt nanoparticles and the alumina support are marked by arrows. b) Comparison of TGA-MS spectra obtained for the Pt and Pt-HP catalysts after the reaction at 540 °C for 6 h. c) The corresponding Raman spectra deconvoluted into several bands, as indicated.

Nonetheless, our finding that a more stable Pt catalyst produces more coke seems counterintuitive. It suggests that the total amount of coke (measured by TGA) does not reflect the catalyst’s long-term stability. In principle, the coke formation may correlate with catalytic activity, which is higher for the Pt–HP catalyst. We propose that the plasma-induced restructuring of the alumina surface, as well as the cleanup of the initial surface from carbon residues,^33^ facilitates the spillover process and provides a higher capacity for the accumulation of carbon over alumina while keeping the Pt surface (modified by carbon) in its most active state.

## 4 Conclusions

Our study demonstrated that the catalyst pretreatment with “cold” plasmas may show considerable beneficial effects on the catalytic performance of low-loaded Pt catalysts in the propane dehydrogenation reaction. In particular, we found that Pt/Al_2_O_3_ catalysts pretreated with a hydrogen plasma before the conventional calcination and reduction steps showed considerably increased propane conversion without loss of selectivity. On the other hand, the catalyst treated with an argon plasma did not display such an effect. The results of the structural characterization using several bulk- and surface-sensitive techniques showed that the H_2_ plasma partially reduces the singly dispersed Pt^2+^ precursor species while promoting metallic Pt clustering, even at near room temperature. In addition, the amount of Cl remaining from the synthesis precursor considerably decreased. Finally, the H_2_ plasma enhances the surface hydroxylation of the alumina support and also modifies the Al^3+^ sites in the same way as a high-temperature treatment in molecular H_2_. In this way, it was possible to realize the intended control of the nucleation and growth of the Pt nanostructures using the Pt SA precursor. Our approach removed the chlorine from the Pt precursor at low temperatures. It also resulted in the fortunate formation of undercoordinated Al sites and the presence of reactive hydroxyl species that provide surface ligands, facilitating the formation of small and strongly anchored Pt nanoparticles, exposing a rough and dynamic surface. Argon plasma also partially removes the chlorine from the precursor but does not create undercoordinated Al sites to stabilize Pt species during high-temperature reduction, thus leading to more three-dimensional nanoparticles.

The present study opens up opportunity for optimizing the application of reactive plasma treatments on low-reactivity precursors to generate transient reactivity. This approach can be used to create a strong functional interface, enabling the preparation of small nanostructures of an active phase with anisotropic (platelet) morphology on a support of catalytic relevance. In this way, the three-layer concept^74^ of catalysts—comprising a reactive surface and a functional surface separated by a subsurface interlayer—can be achieved through wet chemical means, provided that a controllable and scalable plasma treatment process is established. Using the mechanochemical preparation pathway gives access to a range of thermodynamically stable bulk supports that are suitable for transient surface activation. By using doped variants of, e.g., metal oxyhydroxides, one can anticipate not only influencing the conductivity properties of the support but also modifying the reactivity of undercoordinated sites containing dopant species. The combination of synthetic unit operations illustrated in this study provides a method for creating families of supported catalysts with enhanced property control of the active phase compared to the enigmatic impregnation methodologies.

## Supplementary Material



## Data Availability

Data will be made available on request.

## Notes

Schematic diagram of the plasma setup. Propene yield and deactivation rates measured on untreated and plasma-treated catalysts. HAADF-STEM images of the catalysts after various treatments. Additional XANES and EXAFS spectra of the catalysts and their fitting analysis. *In situ* CO DRIFTS spectra (PDF)
