# Supplementary material for: Plasma Pretreatment of Pt Single-Atom Precursors Supported on Mechanically Activated Al2O3: Enhanced Performance in Propane Dehydrogenation
Source: J Am Chem Soc. 2025 Oct 27;147(44):40481–95. doi: 10.1021/jacs.5c12230 (PMC12593335; doi:10.1021/jacs.5c12230)
Supplement: Supplementary file 1 [file ja5c12230_si_001.pdf]

Supporting Information

Plasma Pre-treatment of Pt Single Atom Precursors Supported on Mechanically Activated Al_2O_3: Enhanced Performance in Propane Dehydrogenation

Jingyi Yang,[1] Eduardo Ortega,[1] Joonbaek Jang,[1] Andrea Martini,[1] Jie Zhu,[1] Janis Timoshenko,[1] Jacopo De Bellis,[2] Ferdi Schüth,[2] Shamil Shaikhutdinov,[1]* Beatriz Roldan Cuenya[1]*

[1] *Department of Interface Science, Fritz-Haber-Institut der Max-Planck-Gesellschaft, Faradayweg 4-6, 14195 Berlin*

[2] *Department of Heterogeneous Catalysis, Max-Planck-Institut für Kohlenforschung, Kaiser-Wilhelm-Platz 1, 45470 Mülheim an der Ruhr*

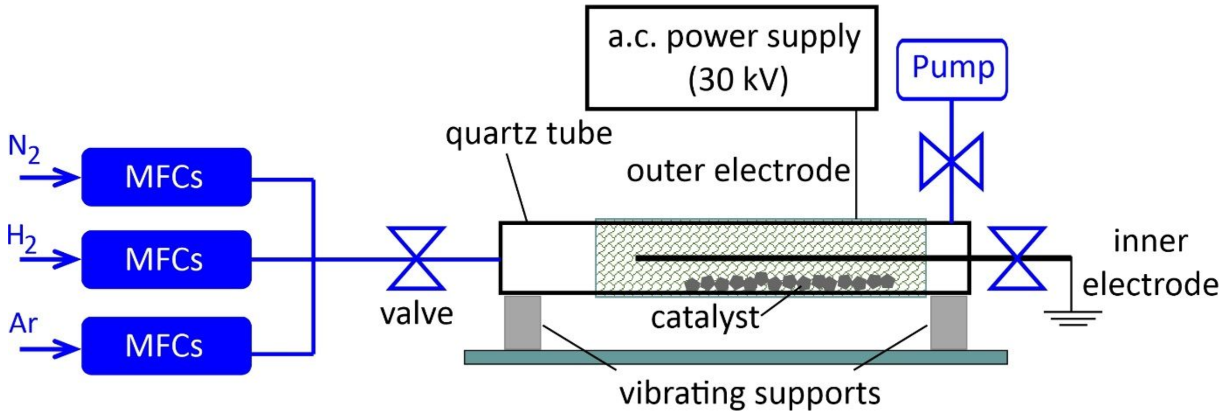

Figure S1. Schematic diagram of the plasma setup. The catalyst powder sample is dispersed onto the wall of a quartz tube (~ 6 mm inner diameter) placed horizontally. During the plasma treatment, the tube was vibrated for the catalyst granules to intermix and hence provide a homogeneous plasma exposure. The inner electrode is silica protected W rod (2 mm in diameter) and electrically grounded. The a.c. voltage (40 kHz, 30 kV) is applied to the outer electrode made of Cu mesh. The current was measured by voltage divider connected to the outer electrode and power supply output. The RMS power was set to ~ 200 W for the plasma treatments. The gas pressure in the plasma is about 5 mbar.

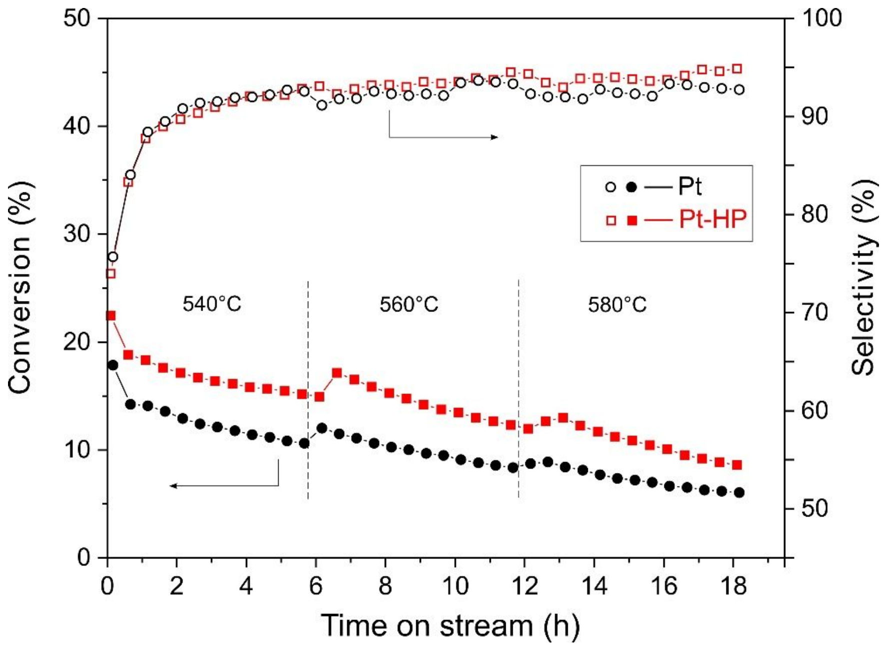

Figure S2. Conversion and selectivity of untreated (Pt) and H_2 plasma-treated (Pt-HP) catalysts as a function of time on stream measured at reaction temperatures increased stepwise as indicated.

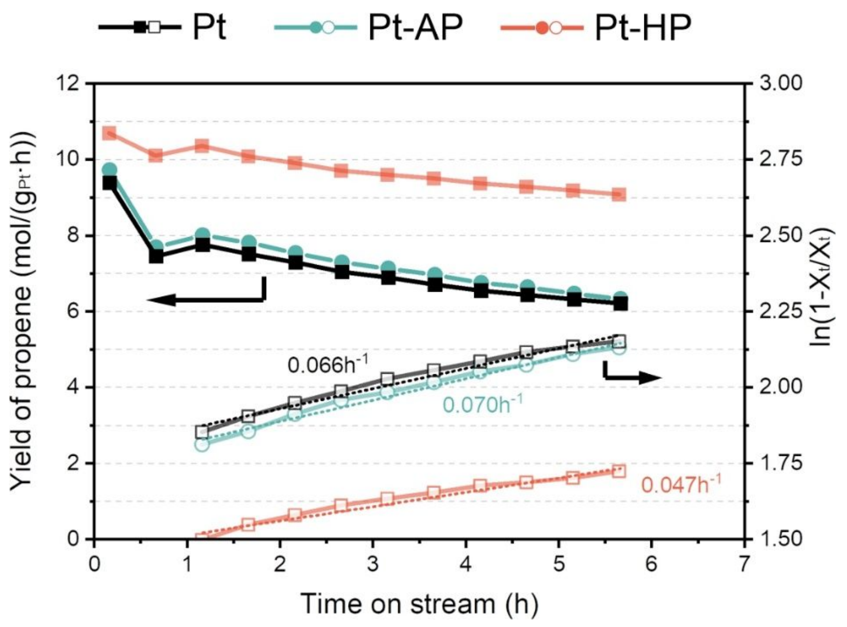

Figure S3. Propene yield and deactivation rate measured on untreated and plasma-treated catalysts as a function of time on stream.

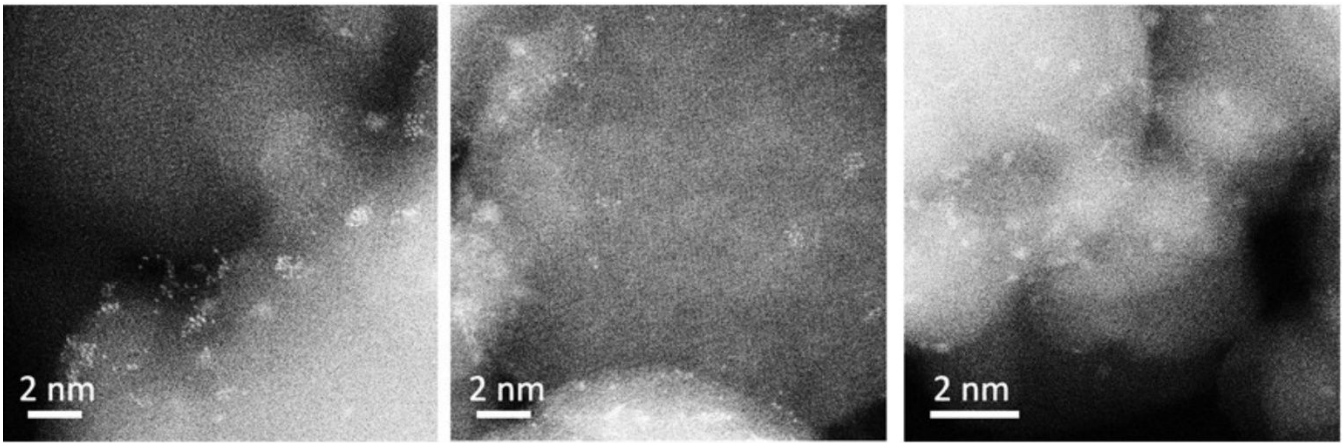

Figure S4. HAADF-STEM images of the 0.2 wt.% Pt/Al_2O_3 catalyst calcined in oxygen at 500°C showing the formation of Pt-oxide clusters and nanoparticles.

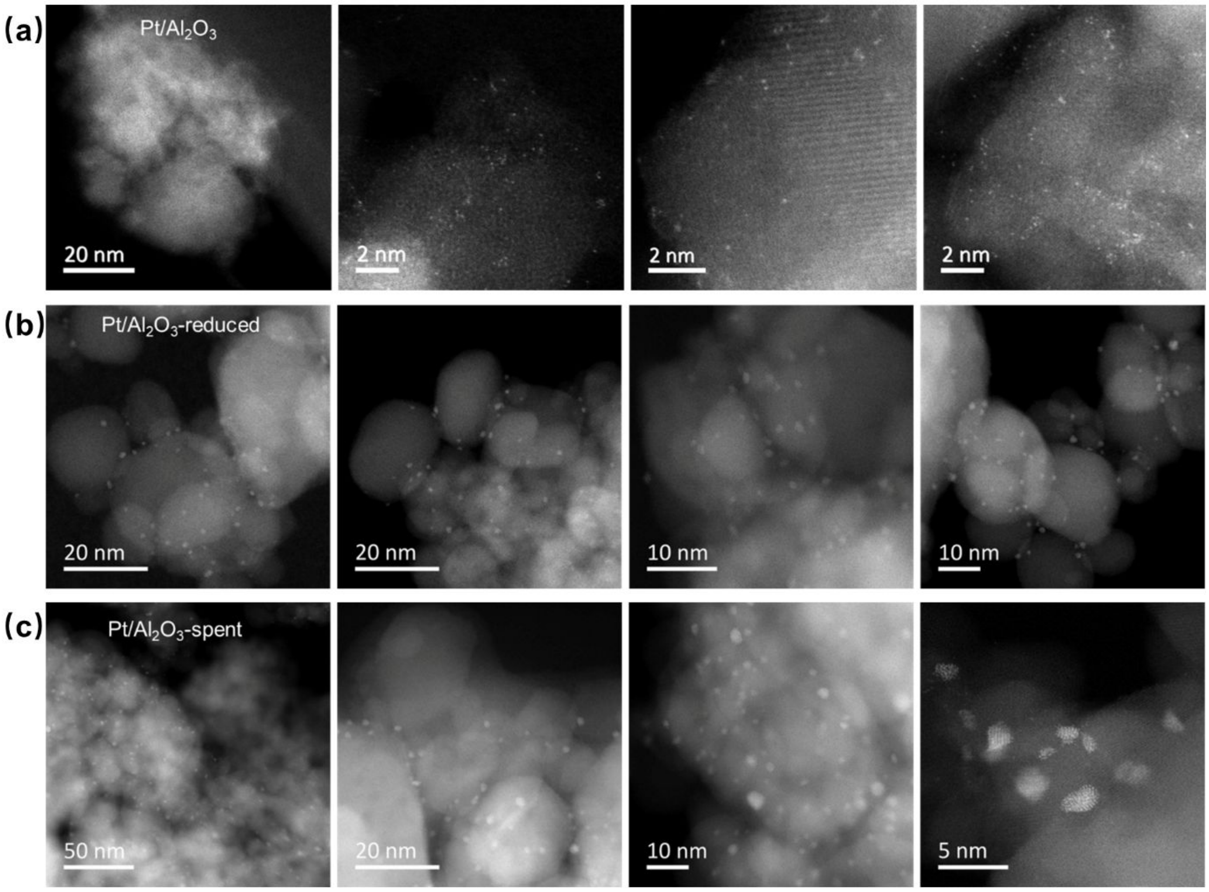

Figure S5. HAADF-STEM images of the 0.2 wt.% Pt/Al_2O_3 catalysts: as prepared (a), after subsequent oxidation at 200°C and reduction in H_2 at 540°C (b), and after 6 hours in the PDH reaction (c). The particle size distribution histogram is shown in Figure 2d in the main text.

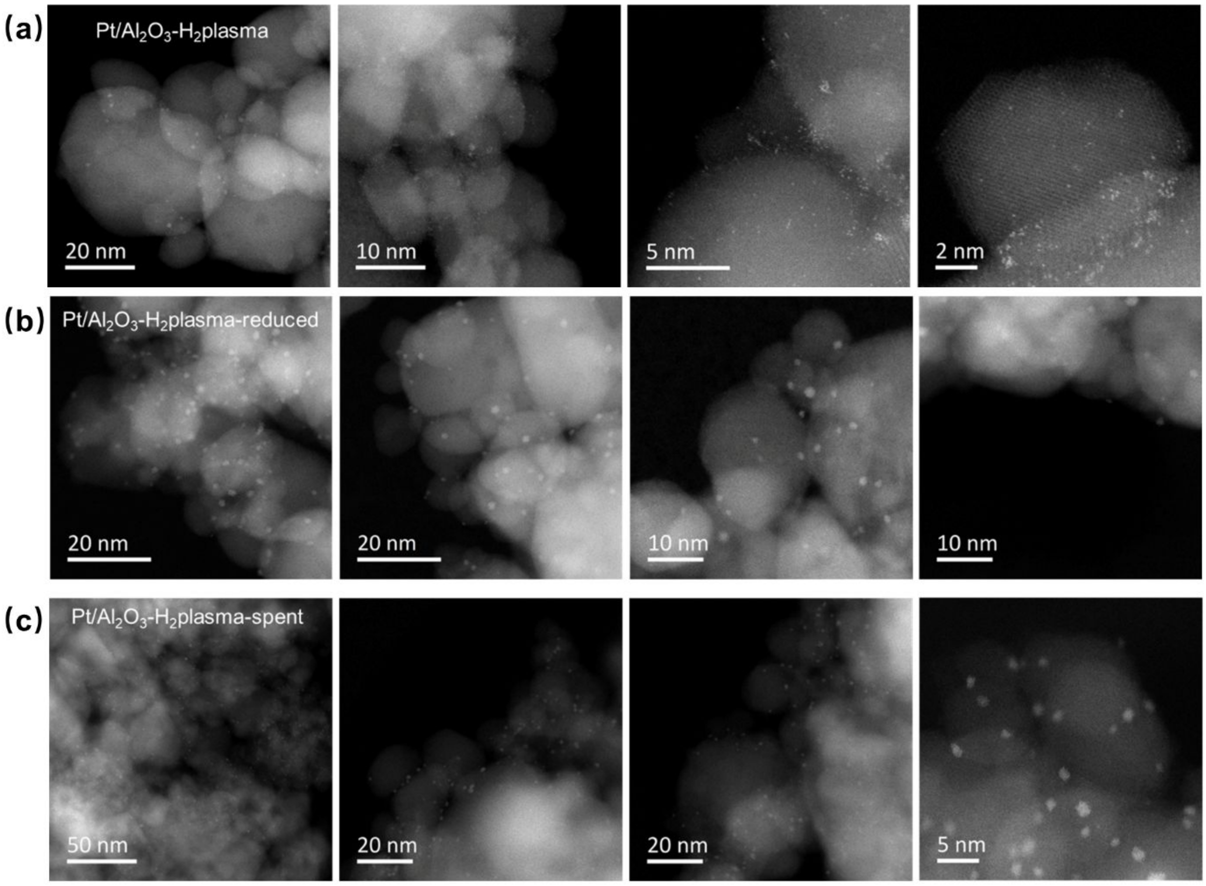

Figure S6. HAADF-TEM images of the 0.2 wt.% Pt/Al_2O_3 catalysts: after H_2 plasma pre-treatment (a), after subsequent oxidation at 200°C and reduction in H_2 at 540°C (b), and after 6 hours in the PDH reaction (c). The particle size distribution histogram is shown in Figure 2h in the main text.

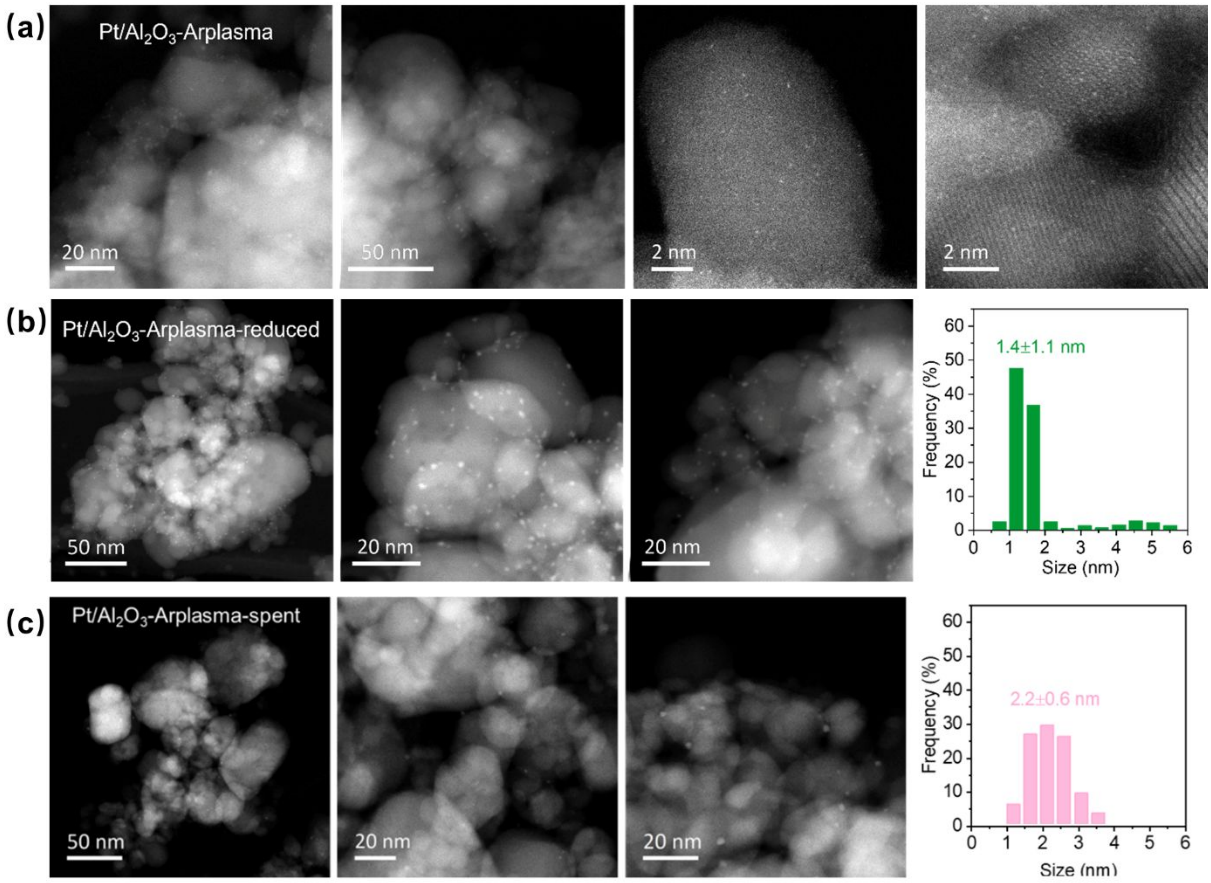

Figure S7. HAADF-TEM images and size histograms of the 0.2 wt.% Pt/Al_2O_3 catalysts pretreated with Ar plasma (a) after subsequent oxidation at 200°C and reduction in H_2 at 540°C (b), and after 6 hours in the PDH reaction (c).

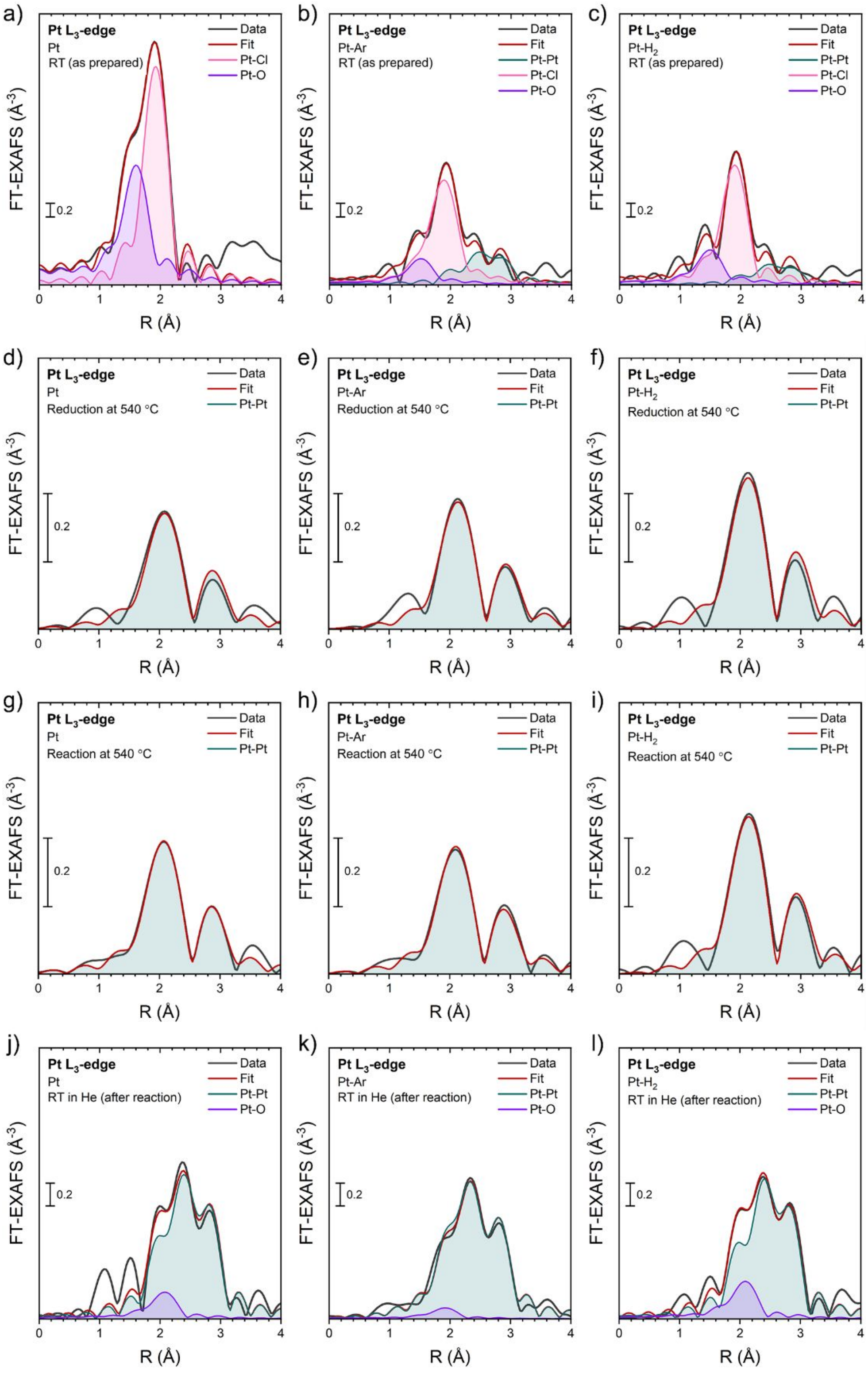

Figure S8. Pt L_3-edge Fourier-transformed (FT)-EXAFS fitting for Pt (a, d, g, j), Pt-AP (b, e, h, k), and Pt-HP (c, f, i, l) catalysts. Measurements were performed: at room temperature on the as-prepared sample (a-c); under reduction in H_2 at 540°C (d-f); during PDH reaction at 540°C (g-i); and at room temperature in He after the reaction (j-l). Experimental EXAFS spectra are compared to their fits. Partial contributions of Pt-Pt, Pt-Cl, and Pt-O scattering paths are shown in different colors.

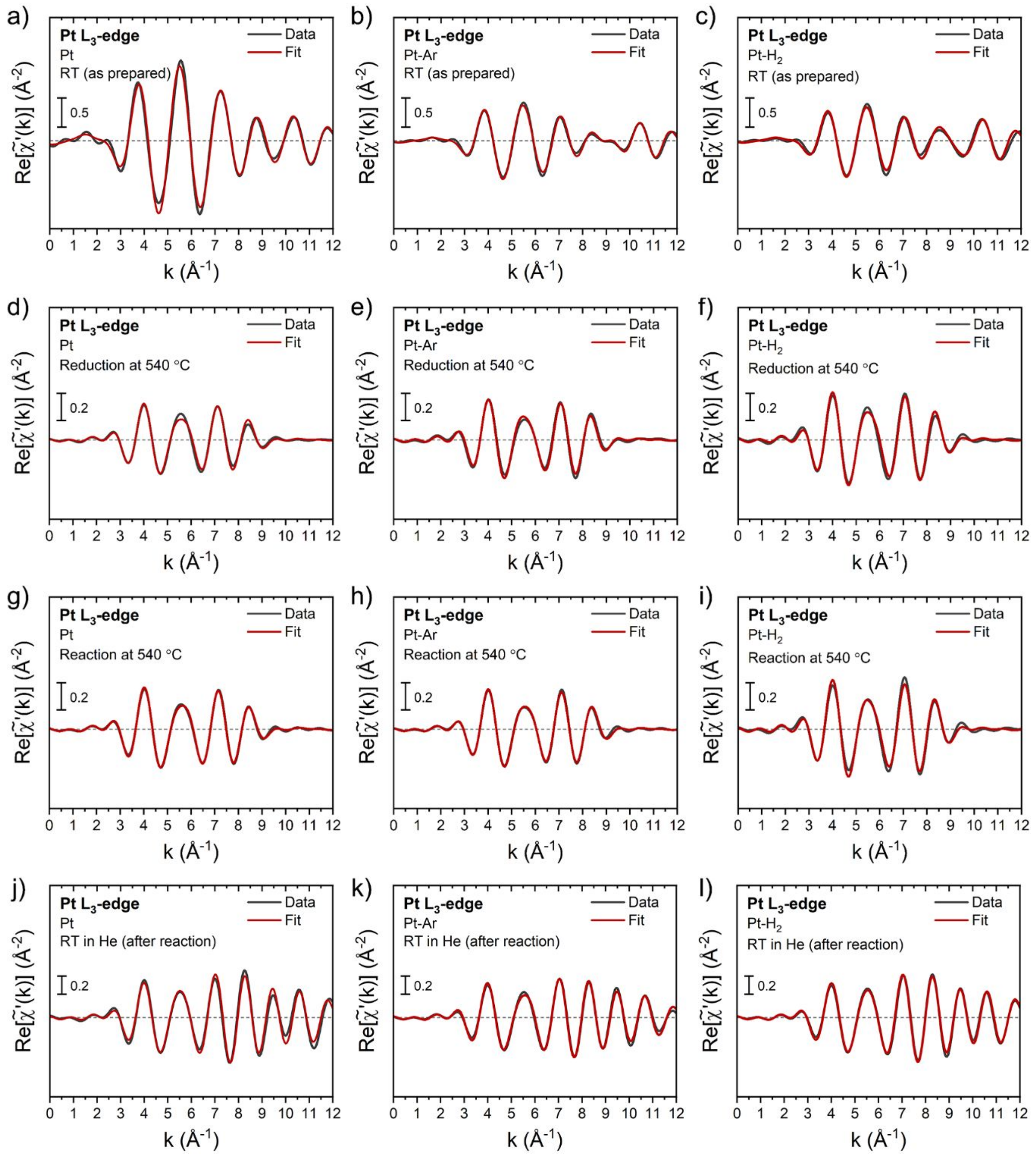

Figure S9. Pt L_3-edge Fourier-filtered EXAFS spectra for Pt (a, d, g, j), Pt-Ar (b, e, h, k), and Pt-H_2 (c, f, i, l) catalysts. Measurements were performed: at room temperature on the as-prepared sample (a-c); under reduction in H_2 at 540°C (d-f); during PDH reaction at 540°C (g-i); and at room temperature in He after the reaction (j-l).

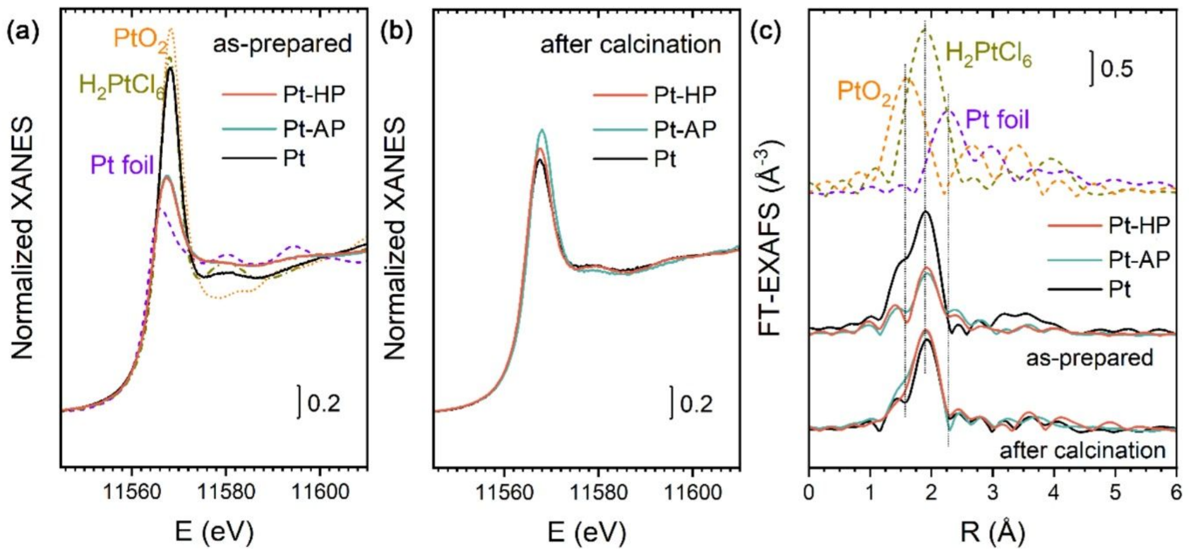

Figure S10. Pt L_3-edge XANES spectra of Pt, Pt-AP, and Pt-HP catalysts: (a) as-prepared, and (b) after calcination at 200°C in O_2. (c) The corresponding FT-EXAFS spectra, compared with the FT-EXAFS data for reference compounds. Spectra are grouped and offset for clarity.

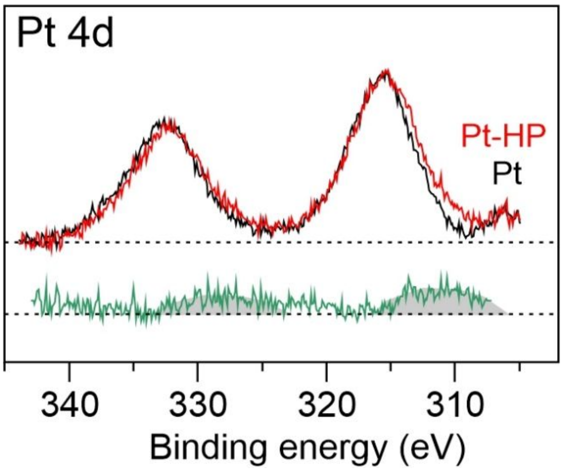

Figure S11. Direct comparison of the Pt 4d spectra obtained for the “as prepared” (Pt) and hydrogen plasma treated (Pt-HP) samples. The difference spectrum is obtained after Shirley type baseline correction implemented in CasaXPS software.

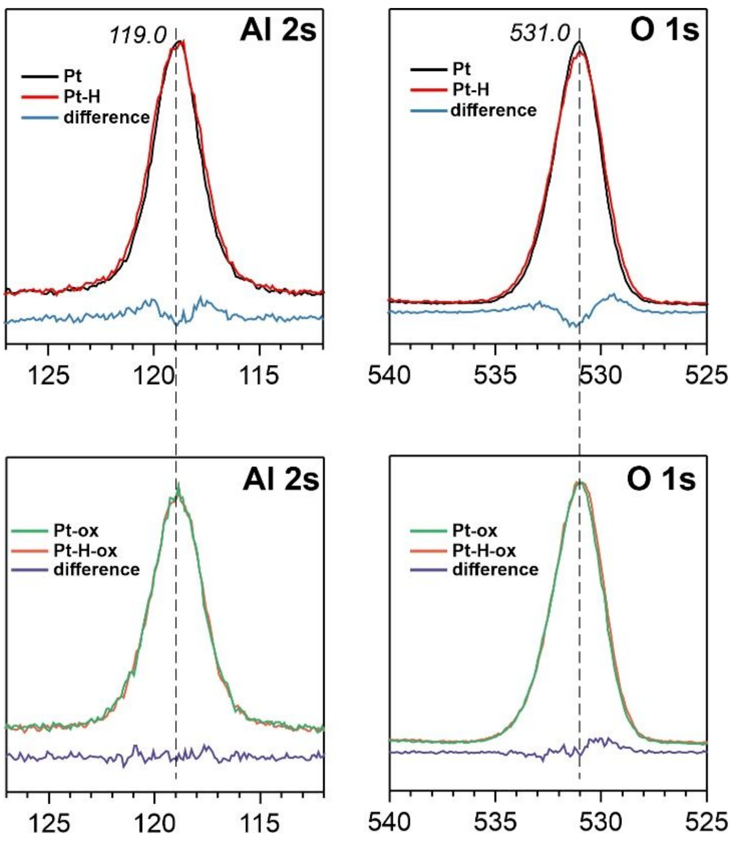

Figure S12. Top panel: Comparison of Al 2s and O 1s XPS spectra obtained for untreated (Pt) and low pressure H_2 plasma treated (Pt-H) catalysts. Bottom panel: The same samples after oxidation in 100 mbar O_2 at 200°C (Pt-ox, and Pt-H-ox, respectively). In both panels, the difference spectra are also shown. The spectra were calibrated using the Al 2s line as internal reference. Note a slight broadening of the Al and O signals upon low pressure plasma treatment which disappears after heating in oxygen.

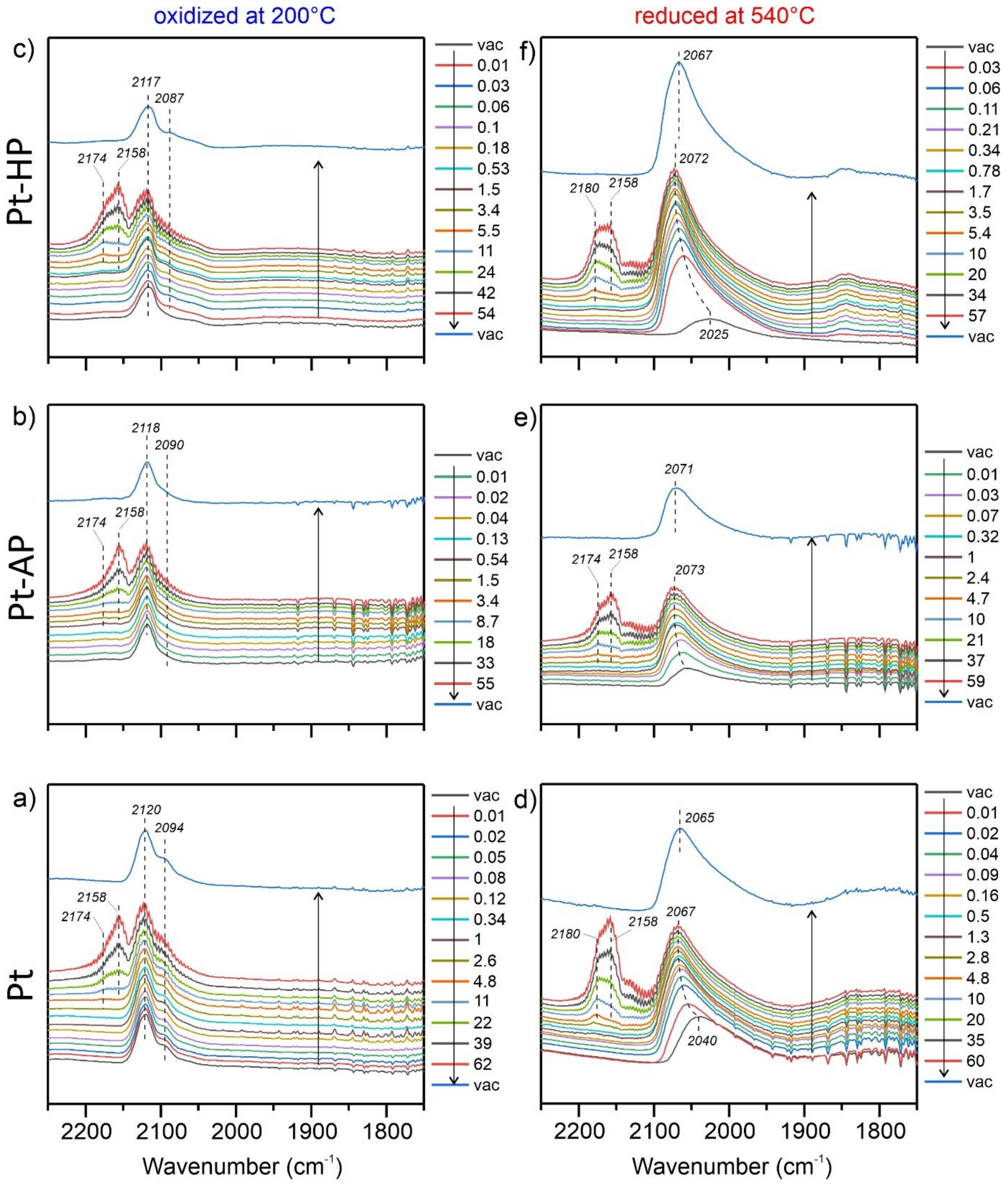

Figure S13. In situ CO DRIFTS spectra obtained for Pt (a,d), Pt-AP (b,e), and Pt-HP (c,f) catalysts after oxidation in O_2 at 200°C (left panel) and after reduction in H_2 at 540°C (right panel). The spectra were recorded at the sample temperature of -135°C with increasing CO pressures and referenced to KBr. Each panel also depicts the spectrum measured in vacuum after final CO exposure. The spectra are offset for clarity. Please note that CO species were present on the samples even before CO dosing.

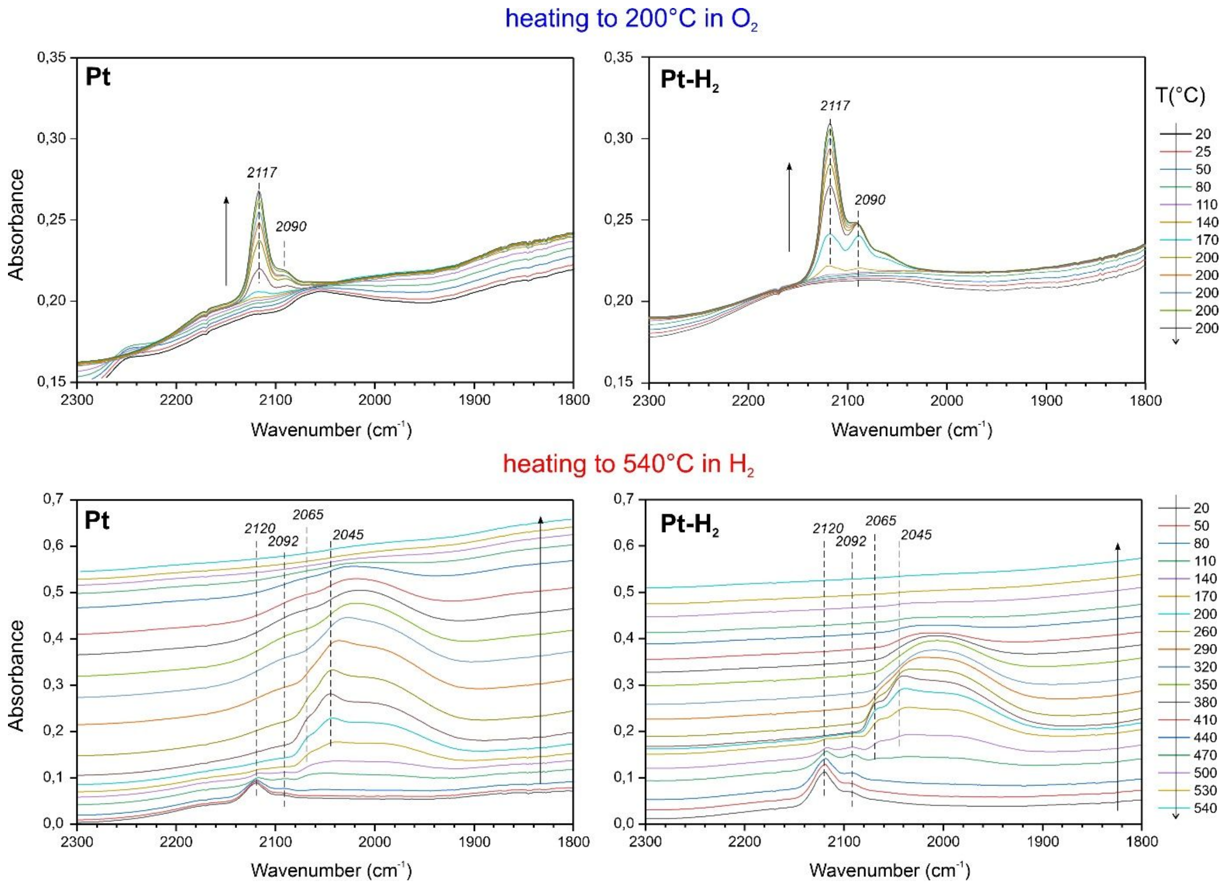

Figure S14. In situ DRIFTS spectra recorded on Pt and Pt-HP catalysts upon heating in O_2 at 200°C (top panel) and in H_2 at 540°C (bottom panel). Some spectra are offset for clarity.

Table S1. Catalytic performance of the Pt catalysts obtained in this work in comparison to an earlier study using the mechanochemically prepared Pt/α-Al_2O_3 catalysts.

Catalyst (Pt loading)	Reaction T (°C)	Initial Rate C_3H_8 (mol/g_{Pt}/h)	6 h TOS Rate C_3H_8 (mol/g_{Pt}/h)	Deactivation Rate* (h^{-1})	Refs.
Pt (0.2)-H2	540	10.6	8.5	0.047	This work
Pt (0.2)-Ar	540	7.7	5.9	0.070	This work
Pt (0.2)	540	8.0	6.0	0.068	This work
Pt (0.14)	500	8.67	6.4**	0.015	Ref. [1]
PtSn (0.09 Pt + 0.05 Sn)	500	13.55	9.9**	0.010	Ref. [1]

* measured for 2 - 6 h of TOS in our work, and 10 - 20 h of TOS in Ref.[1]
** evaluated from Figure 1 in Ref. [1]

Note S1: The synthesis procedure in Ref. [1], using ball-milling of the Pt and alumina physical mixture, and in the present study, applying molecular Pt precursor adsorption, are substantially different. Nonetheless, the agreement between two studies is fairly good and lends support to the statement that the hydrogen plasma treatment improves the catalytic performance for at least the experimental TOS used here.

Table S2. Pt L_3-edge first-shell EXAFS fitting results for Pt, Pt-AP, and Pt-HP catalysts. Fit uncertainties for the correction to photoelectron reference energy (ΔE_0), coordination numbers (CN), and bond lengths (r) are shown in the parentheses, and show the uncertainty in the last digit. The disorder factors (σ^2) were first obtained from the fit, and then fixed to improve the uncertainties of other fitting parameters. Similarly, an average σ^2 was obtained for high-temperature data (using 22 spectra at 540 °C) and applied to all fitting of high-temperature data for consistent estimation of Pt-Pt CNs.

As-prepared sample transferred in Ar, measured at room temperature

		Pt			Pt-Ar			Pt-H_2	
**Fit R-factor**		0.019			0.017			0.038	
**ΔE_0 (eV)**		10 (2)			8 (1)			7 (2)	
	-	Pt-Cl	Pt-O	Pt-Pt	Pt-Cl	Pt-O	Pt-Pt	Pt-Cl	Pt-O
**CN**		2.8 (2)	2.4 (3)	1.7 (4)	2.3 (2)	0.5 (2)	1.1 (5)	2.0 (2)	0.6 (3)
**r (Å)**		2.30 (1)	1.99 (2)	2.76 (1)	2.30 (1)	1.90 (3)	2.76 (2)	2.30 (1)	1.89 (3)
**σ^2 (Å^2)**		0.001	0.002	0.004	0.006	0.002	0.004	0.004	0.002

in situ reduction in H_2 at 540°C

	Pt	Pt-Ar	Pt-H_2
**Fit R-factor**	0.019	0.020	0.024
**ΔE_0 (eV)**	4 (1)	6 (1)	6 (1)
	**Pt-Pt**	**Pt-Pt**	**Pt-Pt**
**CN**	7.7 (5)	8.6 (6)	10.2 (7)
**r (Å)**	2.72 (1)	2.75 (1)	2.75 (1)
**σ^2 (Å^2)**	0.01415	0.01415	0.01415
**C_3 (Å^3)**	0.001	0.001	0.001

PDH reaction at 540°C

	Pt	Pt-Ar	Pt-H_2
**Fit R-factor**	0.005	0.006	0.025
**ΔE_0 (eV)**	3.8 (6)	4.6 (6)	6 (1)
	**Pt-Pt**	**Pt-Pt**	**Pt-Pt**
**CN**	8.7 (3)	8.5 (3)	10.6 (8)
**r (Å)**	2.708 (5)	2.727 (6)	2.75 (1)
**σ^2 (Å^2)**	0.01415	0.01415	0.01415
**C_3 (Å^3)**	0.001	0.001	0.001

After PDH reaction, measured at room temperature in He

	Pt		Pt-Ar		Pt-H_2	
**R-factor**	0.040		0.012		0.009	
**ΔE_0 (eV)**	5 (2)		4 (1)		5.7 (9)	
	**Pt-Pt**	**Pt-O**	**Pt-Pt**	**Pt-O**	**Pt-Pt**	**Pt-O**
**CN**	6.7 (7)	0.7 (4)	8.6 (4)	0.2 (2)	5.8 (3)	0.9 (2)
**r (Å)**	2.73 (1)	2.51 (7)	2.711 (6)	2.36 (6)	2.724 (5)	2.52 (2)
**σ^2 (Å^2)**	0.007	0.002	0.009	0.002	0.006	0.002

Table S3. Assignments of the ν(OH) bands for isolated surface hydroxyls on different aluminas. (Reproduced with permission from ref. [2])

Frequency (cm^{-1})	Tsyganenko [3]	Knözinger [4]	Busca [5]	Digne et al.[6]
3785 - 3800	I	Ib (tetr)	I (tetr)	HO-μ_1-Al_{IV} (110)
3760 - 3780	I	Ia (oct)	I (tetr-vac)	HO-μ_1-Al_{VI} (100)
3745	II	IIb (tetr-oct)		
3730–3735	II	IIa (oct-oct)	I (oct)	HO-μ_2-Al_V (110)
3710	III	III	I (oct-vac)	
3690	III	III	II	HO-μ_1-Al_V (110)
3590	H-bonded	III	II	HO-μ_3-Al_{VI} (100)

I and μ_1, II and μ_2, III and μ_3 denote terminal, bridging, and triply bonded OH, respectively;
tetr (oct) and Al_{IV} (Al_{VI}) denote OH bonded to tetrahedral and octahedral Al ions, respectively;
vac. denotes vacancy nearby;
(110) and (100) are different planes of γ-Al_2O_3

Note S3: The assignments of ν(OH) bands on Al_2O_3 remain controversial. The ν(OH) bands at 3790 and 3730 cm^{-1} are commonly assigned to terminal OH's on tetrahedral and octahedral Al cations, respectively, the band at ~ 3770 cm^{-1} can equally be attributed to OH bonded to a penta-coordinated (Al_V) cation in an unstable coordination state(from DFT study[6]) or to OH located near a cation vacancy.[5] The latter was also supported by another DFT study[7] that assigned the 3775 cm^{-1} band to defective Al_{IV} sites.

Table S4. Assignment of ν(CO) bands on Al_2O_3 (from refs. [7-9]) and on Pt (from refs. [10-17]).

**Average frequency (cm^{-1})**	**CO/Al_2O_3**
> 2210 (up to 2240)	Al_{IV} sites in crystallographically defective configuration; Al_{III} sites
2210–2195	Al_{IV} sites on extended facets of regular low-index crystal planes
2185 - 2175	$[Al^{VI}]_{IV}$ sites on the main faces
2165–2150	$[Al^{VI}]_{V}$ sites
~ 2155	CO on surface OH
	**CO/Pt**
2120 - 2100	Single $Pt^{\delta+}$ atoms; oxidized Pt
2090 - 2100	Single Pt^0 atoms; surface Pt^+ atoms
2090 - 2080	High-coordination (terrace) sites on NPs
2070 - 2030	Low-coordination (edge) sites on NPs
2030 - 2010	Corners, kinks on NPs
1880 - 1830	Bridged CO on terrace (edge) sites

Note S4a: FTIR studies of CO on several aluminas revealed a significant heterogeneity of Al^{3+} Lewis acid sites at the surface. In general, the highest frequency bands are associated with CO adsorbed on the most uncoordinated sites such as tri-coordinated Al_{III}.[2] CO interacting with penta-coordinated Al_V gives rise to the ν(CO) bands at 2150–2160 cm^{-1}, while CO interacting with tetra-coordinated Al_{IV} - at 2210 - 2180 cm^{-1}.

The spectra on α-, γ-, and δ-Al_2O_3 revealed similar features, albeit of different intensities, showing the ν(CO) bands at 2184 - 2173 and 2163 - 2153 cm^{-1}, which were assigned to CO adsorbed on $[Al^{VI}]_{IV}$ and $[Al^{VI}]_{V}$ sites, respectively, on the main exposed faces. The respective bands for CO adsorbed on coordinatively unsaturated Al cations located on defects seems to appear at higher frequency, i.e., at 2230 and 2215 cm^{-1} for corner and edges/steps sites, respectively.[8]

The highest frequency bands (> 2210 cm^{-1}) and the band at 2210 – 2195 cm^{-1} are associated with CO adsorbed on Al_{IV} sites in crystallographically defective configuration and on extended facets of regular low-index crystal planes, respectively.[7]

On partially hydroxylated alumina surfaces, the band at ~ 2155 cm^{-1} is dominated by H-bonded CO species.[7, 9]

Note S4b: As followed from the DFT study[15] performed for CO adsorption on unsupported Pt clusters, a unique wavenumber cannot be assigned straightforwardly to a specific binding site.

The band position depends not only on the interaction of CO with the Pt site, but also on the degree of dipole–dipole interaction with neighboring CO adsorbed molecules, and also on total CO coverage. This renders the precise band assignment somewhat ambiguous.

References

1. Pfister, N.; Kraievska, I.; Rohner, C.; Dong, J.; Timpe, O.; Girgsdies, F.; Lunkenbein, T.; Khobragade, R.; De Bellis, J.; Schüth, F.; Trunschke, A., A Facile Approach to Alumina-Supported Pt Catalysts for the Dehydrogenation of Propane. *Industrial & Engineering Chemistry Research* **2024,** *63* (48), 20778-20786.
2. Busca, G., The surface of transitional aluminas: A critical review. *Catalysis Today* **2014,** *226*, 2-13.
3. Tsyganenko, A. A.; Filimonov, V. N., Infrared Spectra of Surface Hydroxyl Groups and Crystalline Structure of Oxides. *Spectroscopy Letters* **1972,** *5* (12), 477-487.
4. Knözinger, H.; Ratnasamy, P., Catalytic Aluminas: Surface Models and Characterization of Surface Sites. *Catalysis Reviews* **1978,** *17* (1), 31-70.
5. Busca, G.; Lorenzelli, V.; Ramis, G.; Willey, R. J., Surface sites on spinel-type and corundum-type metal oxide powders. *Langmuir* **1993,** *9* (6), 1492-1499.
6. Digne, M.; Sautet, P.; Raybaud, P.; Euzen, P.; Toulhoat, H., Use of DFT to achieve a rational understanding of acid–basic properties of γ-alumina surfaces. *Journal of Catalysis* **2004,** *226* (1), 54-68.
7. Morterra, C.; Magnacca, G., A case study: surface chemistry and surface structure of catalytic aluminas, as studied by vibrational spectroscopy of adsorbed species. *Catalysis Today* **1996,** *27* (3), 497-532.
8. Gribov, E. N.; Zavorotynska, O.; Agostini, G.; Vitillo, J. G.; Ricchiardi, G.; Spoto, G.; Zecchina, A., FTIR spectroscopy and thermodynamics of CO and H2 adsorbed on γ-, δ- and α-Al2O3. *Physical Chemistry Chemical Physics* **2010,** *12* (24), 6474-6482.
9. Szanyi, J.; Kwak, J. H., Dissecting the steps of CO2 reduction: 1. The interaction of CO and CO2 with γ-Al2O3: an in situ FTIR study. *Physical Chemistry Chemical Physics* **2014,** *16* (29), 15117-15125.
10. O'Brien, C. P.; Jenness, G. R.; Dong, H.; Vlachos, D. G.; Lee, I. C., Deactivation of Pt/Al2O3 during propane oxidation at low temperatures: Kinetic regimes and platinum oxide formation. *Journal of Catalysis* **2016,** *337*, 122-132.
11. Kaftan, A.; Kusche, M.; Laurin, M.; Wasserscheid, P.; Libuda, J., KOH-promoted Pt/Al2O3 catalysts for water gas shift and methanol steam reforming: An operando DRIFTS-MS study. *Applied Catalysis B-Environmental* **2017,** *201*, 169-181.
12. Morfin, F.; Dessal, C.; Sangnier, A.; Chizallet, C.; Piccolo, L., Identification of Stable Species Formed Under CO Adsorption and Oxidation on Alumina-Supported Single Pt Atoms: Why Nanoparticles Are More Active. *Acs Catalysis* **2024,** *14* (12), 9628-9639.
13. Zhang, Z.; Zhu, Y.; Asakura, H.; Zhang, B.; Zhang, J.; Zhou, M.; Han, Y.; Tanaka, T.; Wang, A.; Zhang, T.; Yan, N., Thermally stable single atom Pt/m-Al2O3 for selective hydrogenation and CO oxidation. *Nature Communications* **2017,** *8* (1), 16100.
14. Carosso, M.; Fovanna, T.; Ricchebuono, A.; Vottero, E.; Manzoli, M.; Morandi, S.; Pellegrini, R.; Piovano, A.; Ferri, D.; Groppo, E., Gas phase vs. liquid phase: monitoring H2 and CO adsorption phenomena on Pt/Al2O3 by IR spectroscopy. *Catalysis Science & Technology* **2022,** *12* (4), 1359-1367.
15. Lentz, C.; Jand, S. P.; Melke, J.; Roth, C.; Kaghazchi, P., DRIFTS study of CO adsorption on Pt nanoparticles supported by DFT calculations. *Journal of Molecular Catalysis A: Chemical* **2017,** *426*, 1-9.
16. de Ménorval, L.-C.; Chaqroune, A.; Coq, B.; Figueras, F., Characterization of mono- and bi-metallic platinum catalysts using CO FTIR spectroscopy Size effects and topological segregation. *Journal of the Chemical Society, Faraday Transactions* **1997,** *93* (20), 3715-3720.

17. Brandt, R. K.; Hughes, M. R.; Bourget, L. P.; Truszkowska, K.; Greenler, R. G., The interpretation of CO adsorbed on Pt/SiO2 of two different particle-size distributions. *Surface Science* **1993,** *286* (1), 15-25.
